# Genome-wide association study of red blood cell traits in Hispanics/Latinos: The Hispanic Community Health Study/Study of Latinos

**DOI:** 10.1371/journal.pgen.1006760

**Published:** 2017-04-28

**Authors:** Chani J. Hodonsky, Deepti Jain, Ursula M. Schick, Jean V. Morrison, Lisa Brown, Caitlin P. McHugh, Claudia Schurmann, Diane D. Chen, Yong Mei Liu, Paul L. Auer, Cecilia A. Laurie, Kent D. Taylor, Brian L. Browning, Yun Li, George Papanicolaou, Jerome I. Rotter, Ryo Kurita, Yukio Nakamura, Sharon R. Browning, Ruth J. F. Loos, Kari E. North, Cathy C. Laurie, Timothy A. Thornton, Nathan Pankratz, Daniel E. Bauer, Tamar Sofer, Alex P. Reiner

**Affiliations:** 1 Department of Epidemiology, University of North Carolina Gillings School of Public Health, Chapel Hill, NC, United States of America; 2 Department of Biostatistics, University of Washington, Seattle, WA, United States of America; 3 The Charles Bronfman Institute for Personalized Medicine, Icahn School of Medicine at Mount Sinai, New York, New York, United States of America; 4 The Genetics of Obesity and Related Metabolic Traits Program, The Icahn School of Medicine at Mount Sinai, New York, New York, United States of America; 5 Division of Public Health Sciences, Fred Hutchinson Cancer Research Center, Seattle, WA, United States of America; 6 New York Genome Center, New York, NY, United States of America; 7 Division of Hematology/Oncology, Boston Children's Hospital, Boston, MA, United States of America; 8 School of Medicine, Wake Forest University, Winston-Salem, NC, United States of America; 9 Joseph J. Zilber School of Public Health, University of Wisconsin Milwaukee, Milwaukee, WI, United States of America; 10 Institute for Translational Genomics and Population Sciences, Los Angeles Biomedical Research Institute at Harbor-UCLA Medical Center, Torrance, CA United States of America; 11 Department of Pediatrics, Los Angeles Biomedical Research Institute at Harbor-UCLA Medical Center, Torrance, CA United States of America; 12 Department of Medicine, University of Washington, Seattle, WA United States of America; 13 Department of Genetics, University of North Carolina, Chapel Hill, NC, United States of America; 14 Department of Biostatistics, University of North Carolina, Chapel Hill, NC, United States of America; 15 Department of Computer Science, University of North Carolina, Chapel Hill, NC, United States of America; 16 Division of Cardiovascular Sciences, National Heart, Lung, and Blood Institute, Bethesda, MD, United States of America; 17 Research and Development Department, Central Blood Institute, Blood Service Headquarters, Japanese Red Cross Society, Tokyo, Japan; 18 Cell Engineering Division, RIKEN BioResource Center, Tsukuba, Ibaraki, Japan; 19 Comprehensive Human Sciences, Tsukuba University, Tsukuba, Ibaraki, Japan; 20 The Mindich Child Health and Development Institute, Icahn School of Medicine at Mount Sinai, New York, New York, United States of America; 21 Division of Laboratory Medicine & Pathology, University of Minnesota, Minneapolis, MN, United States of America; 22 Department of Pediatric Oncology, Dana-Farber Cancer Institute, Boston, MA, United States of America; 23 Department of Pediatrics, Harvard Medical School and Harvard Stem Cell Institute, Harvard University, Boston, MA, United States of America; Case Western Reserve University School of Medicine, UNITED STATES

## Abstract

Prior GWAS have identified loci associated with red blood cell (RBC) traits in populations of European, African, and Asian ancestry. These studies have not included individuals with an Amerindian ancestral background, such as Hispanics/Latinos, nor evaluated the full spectrum of genomic variation beyond single nucleotide variants. Using a custom genotyping array enriched for Amerindian ancestral content and 1000 Genomes imputation, we performed GWAS in 12,502 participants of Hispanic Community Health Study and Study of Latinos (HCHS/SOL) for hematocrit, hemoglobin, RBC count, RBC distribution width (RDW), and RBC indices. Approximately 60% of previously reported RBC trait loci generalized to HCHS/SOL Hispanics/Latinos, including African ancestral alpha- and beta-globin gene variants. In addition to the known 3.8kb alpha-globin copy number variant, we identified an Amerindian ancestral association in an alpha-globin regulatory region on chromosome 16p13.3 for mean corpuscular volume and mean corpuscular hemoglobin. We also discovered and replicated three genome-wide significant variants in previously unreported loci for RDW (*SLC12A2* rs17764730, *PSMB5* rs941718), and hematocrit (*PROX1* rs3754140). Among the proxy variants at the *SLC12A2* locus we identified rs3812049, located in a bi-directional promoter between *SLC12A2* (which encodes a red cell membrane ion-transport protein) and an upstream anti-sense long-noncoding RNA, *LINC01184*, as the likely causal variant. We further demonstrate that disruption of the regulatory element harboring rs3812049 affects transcription of *SLC12A2* and *LINC01184* in human erythroid progenitor cells. Together, these results reinforce the importance of genetic study of diverse ancestral populations, in particular Hispanics/Latinos.

## Author summary

Red blood cells (RBC) are important for transport of oxygen to tissues throughout the body. Distribution of RBC traits differs by ethnicity and gender, and both genetic and acquired factors likely contribute to these differences. Prior genetic studies have identified physical regions of the genome associated with RBC traits in populations with European, African, and Asian ancestry. These studies have not included individuals with ancestry from the American continents (Amerindian ancestry), such as Hispanics/Latinos. In an analysis of RBC traits in up to 19,608 Hispanics/Latinos, we identified an Amerindian-ancestry genetic association in a known alpha-globin regulatory region. We also identified three new RBC trait associations, including a regulatory variant of *SLC12A2* that encodes a RBC membrane ion-transport protein. Experimental disruption of this regulatory element led to reduced expression of both *SLC12A2* and an adjacent long non-coding RNA in human erythroid progenitor cells. These results contribute to understanding the physiology of red blood cells and reinforce the importance of genetic study of diverse ancestry populations, in particular Hispanics/Latinos.

## Introduction

Red blood cell (RBC) development and maintenance are critical for transport of oxygen to tissues throughout the body. Several parameters commonly measured in clinical blood count evaluations are used to characterize RBC: hematocrit (HCT), hemoglobin (HGB), RBC count, mean corpuscular hemoglobin (MCH), mean corpuscular hemoglobin concentration (MCHC), mean corpuscular volume (MCV), and red cell distribution width (RDW) (detailed trait description provided in **S1 Table**). RBC traits differ by self-reported ancestry, and both genetic (e.g., inherited hemoglobin variants) and acquired (e.g., iron deficiency, kidney disease) factors contribute to these ethnic differences[1, 2]. Quantitative RBC parameters are also polygenic traits that exhibit moderate to high heritability (trait-specific h^2^ between 40% and 90%)[3–5]. Over 80 genomic regions have been associated with one or more RBC traits through genome-wide association studies (GWAS), performed primarily in European- and, to a lesser extent, Asian- and African-descent populations[6–14].

Hispanics/Latinos are ethnically heterogeneous, with admixture of European, West African, and Amerindian ancestral populations. In general, RBC trait values among Hispanics/Latinos have been reported to be similar to those among non-Hispanic whites, though certain types of congenital and acquired anemias are more common among Hispanics/Latinos[15–19]. As with most complex traits, GWAS for discovery or generalization of RBC trait loci has yet to be performed in Hispanics/Latinos or other populations with Amerindian ancestry. In the current study, we performed genome-wide association analysis of seven quantitative RBC traits in 12,502 participants ascertained by the Hispanic Community Health Study/Study of Latinos (HCHS/SOL) and replicated any new association findings discovered in HCHS/SOL in three independent samples of Hispanic/Latino Americans.

## Results

The demographic characteristics and RBC trait distributions of the 12,502 Hispanic/Latino HCHS/SOL participants are summarized in **S2 Table**. Genomic inflation factors for the seven RBC traits ranged from 1.015 (MCHC) to 1.054 (RDW), indicating adequate control of population stratification (**S1 Table**). Overall, 24 loci were significantly associated with one or more RBC traits in HCHS/SOL (**Table 1 and S1 and S2 Figs**). The number of distinct genomic regions associated with each trait were 4 loci for HCT, 4 for HGB, 6 for RBC count, 8 for RDW, 9 for MCH, 5 for MCHC, and 9 for MCV. Association results and allele frequencies of lead SNPs for each genome-wide-significant trait-locus association are presented for six genetic subgroups comprising the HCHS/SOL study population in **S3 Table**.

**Table 1:** Genetic variants significantly associated with red blood cell traits in HCHS/SOL Hispanics/Latinos.

Trait	Status	Annotated Gene(s) (annotation)	rsID/CNV	chr: position	CA	oevar	CAF	Beta (SE)	p-value	1000 Genomes Allele Frequencies				
										AFR	AMR	EAS	SAS	EUR
HCT	**Novel**	***PROX1* (intronic)**	**rs3754140**	**chr1: 214003037**	**T**	**1.03**	**0.61**	**-0.24 (0.05)**	**5.7x10**^**-8**^	**0.71**	**0.55**	**0.69**	**0.83**	**0.73**
	Known	*PRKCE* (intronic)	rs17034641	chr2: 46372644	G	1.00	0.86	0.36 (0.06)	2.6x10^-9^	0.79	0.87	0.94	0.79	0.85
	Known	*HBB* (missense)	rs334	chr11: 5248232	T	0.86	0.99	1.32 (0.20)	1.3x10^-10^	0.90	>0.99	1.00	1.00	1.00
	Known	*TMPRSS6* (missense)	rs855791	chr22: 37462936	A	1.03	0.44	-0.38 (0.04)	1.1x10^-10^	0.10	0.51	0.57	0.54	0.39
HGB	Known	*PRKCE* (intronic)	rs17034641	chr2: 46372644	G	1.00	0.86	0.12 (0.02)	3.2x10^-8^	0.79	0.87	0.94	0.79	0.85
	Known	*HFE* (intronic)	rs2032451	chr6: 26092170	G	1.01	0.88	-0.12 (0.02)	3.1x10^-8^	0.99	0.88	0.97	0.93	0.83
	Known	*HBA1 / HBA2* (intergenic)	3.8kb del ^d^	chr16: 223447	3.8kb del	NA	0.04	-0.46 (0.04)	1x10^-32^	0.16	0.02	0.02	0.02	0.004
	Known	*TMPRSS6* (missense)	rs855791	chr22: 37462936	A	1.03	0.44	-0.15 (0.02)	6.0 x10^-23^	0.10	0.51	0.57	0.54	0.39
RBC Count	Known	*KIT* (intergenic)	rs218265	chr4: 55408999	T	1.07	0.67	0.033 (0.01)	3.6x10^-10^	0.75	0.66	0.65	0.73	0.85
	Known	*HBS1L/MYB* (intergenic)	rs34164109	chr6: 135100038	C	1.00	0.84	0.054 (0.01)	3.6x10^-17^	0.86	0.84	0.76	0.89	0.74
	Known	*TFR2* (intronic)	rs2075672	chr7: 100642673	A	1.03	0.30	0.028 (0.01)	1.4x10^-8^	0.34	0.29	0.23	0.33	0.38
	Known	*HBA1 / HBA2* (intergenic)	3.8kb del ^d^	chr16: 223447	3.8kb del	NA	0.04	0.29 (0.01)	4.4x10^-136^	0.16	0.02	0.02	0.02	0.004
	**Novel**	***RBFOX3* (intronic)**	**rs76539504**	**chr17: 79139365**	**T**	**1.02**	**0.96**	**0.066 (0.01)**	**1.4x10**^**-8**^	**0.81**	**0.96**	**1.00**	**0.99**	**0.97**
	Known^e^	*G6PD* (missense)	rs1050828	chrX: 153764217	C	1.04	0.98	0.13 (0.01)	1.80x10^-18^	0.87	0.99	1.00	1.00	1.00
RDW	**Novel**	**N/A (intergenic)**	**rs6685034**	**chr1: 193954300**	**C**	**1.02**	**0.03**	**-0.02 (0.003)**	**4.8x10**^**-8**^	**0.15**	**0.01**	**0.00**	**0.00**	**0.00**
	Known^a^	*SLC12A7* (intronic)	rs4565255	chr5: 1109568	T	0.98	0.60	0.007 (0.001)	3.1x10^-10^	0.71	0.63	0.69	0.57	0.42
	**Novel**	***SLC12A2* (promoter)**	**rs17764730**	**chr5: 127357526**	**T**	**1.02**	**0.16**	**-0.011 (0.001)**	**8.8x10**^**-13**^	**0.02**	**0.16**	**0.35**	**0.37**	**0.21**
	**Novel**	***PSMB5* (intronic)**	**rs7147308**	**chr14: 23497629**	**C**	**1.10**	**0.70**	**-0.007 (0.001)**	**5.8x10**^**-9**^	**0.13**	**0.79**	**0.94**	**0.60**	**0.70**
	**Novel**	***MCTP2* (intergenic)**	**rs111473449**	**chr15: 95330055**	**G**	**0.99**	**0.97**	**-0.018 (0.003)**	**3.2x10**^**-8**^	**0.84**	**0.97**	**1.00**	**1.00**	**>0.99**
	Known	*HBA1 / HBA2* (intergenic)	3.8kb del ^d^	chr16: 223447	3.8kb del	NA	0.04	0.05 (0.00)	2.4x10^-70^	0.16	0.02	0.02	0.02	0.004
	Known^a^	*TMPRSS6* (missense)	rs855791	chr22: 37462936	A	1.03	0.44	0.007 (0.001)	2.7x10^-11^	0.10	0.51	0.57	0.54	0.39
	Known^e^	*G6PD* (missense)	rs1050828	chrX: 153764217	C	1.04	0.98	0.04 (0.003)	1.50x10^-29^	0.87	0.99	1.00	1.00	1.00
MCH	Known	*TFRC* (intergenic)	rs12634180^c^	chr3: 195825756	G	0.81	0.82	-0.22 (0.04)	2.0x10^-8^	0.91	0.79	NA	NA	0.81
	Known	*KIT* (intergenic)	rs218265	chr4: 55408999	T	1.07	0.67	-0.21 (0.03)	3.3x10^-12^	0.75	0.66	0.65	0.73	0.85
	Known	*HFE* (intronic)	rs2032451	chr6: 26092170	G	1.01	0.88	-0.29 (0.04)	3.5x10^-12^	0.99	0.88	0.97	0.93	0.83
	Known	*CCND3* (intronic)	rs9367125	chr6:41987544	G	0.99	0.92	0.29 (0.05)	1.3x10^−8^	0.96	0.94	0.74	0.86	0.88
	Known	*HBS1L / MYB* (intergenic)	rs9389268	chr6: 135419631	A	1.00	0.83	-0.22 (0.04)	7.9x10^-10^	0.78	0.84	0.76	0.89	0.74
	Known	*CITED2* (intergenic)	rs607203	chr6: 139841653	T	1.02	0.07	0.33 (0.06)	1.7x10^-9^	0.24	0.05	0.00	0.02	0.04
	Known	*HBA1 / HBA2* (intergenic)	3.8kb del ^d^	chr16: 223447	3.8kb del	NA	0.04	-2.60 (0.06)	<2.5x10^-231^	0.16	0.02	0.02	0.02	0.004
	Known	*TMPRSS6* (missense)	rs855791	chr22: 37462936	A	1.03	0.44	-0.34 (0.03)	1.0x10^-34^	0.10	0.51	0.57	0.54	0.39
	Known^e^	*CTAG2 / GAB3* (intergenic)	rs146474788	chrX: 153893403	G	1.04	0.98	-0.56 (0.08)	1.50x10^-29^	0.85	0.99	>0.99	1.00	1.00
MCHC	Known	*SMIM19* (intergenic)	rs1349471	chr8: 42598868	C	1.05	0.44	-0.11 (0.02)	3.0x10^-11^	0.17	0.48	0.43	0.35	0.41
	Known	*HBB* (missense)	rs334	chr11: 5248232	T	0.86	0.99	0.67 (0.08)	3.6x10^-16^	0.90	>0.99	1.00	1.00	1.00
	Known^b^	*HBB* (missense)	rs33930165 ^b^	chr11: 5248233	C	0.85	0.997	-1.86 (0.18)	6.8 x10^-24^	0.99	1.00	1.00	1.00	1.00
	Known	*HBA1 / HBA2* (intergenic)	3.8kb del ^d^	chr16: 223447	3.8kb del	NA	0.04	-0.82 (0.04)	6.7x10^-81^	0.16	0.02	0.02	0.02	0.004
	Known	*PIEZO1* (enhancer)	rs551118	chr16: 88789676	C	0.96	0.48	0.14 (0.02)	3.9x10^-14^	0.26	0.50	0.38	0.40	0.41
	Known	*KCTD17* (enhancer)	rs9610638	chr22: 37049628	T	1.00	0.43	-0.14 (0.02)	7.0x10^-17^	0.06	0.49	0.58	0.56	0.39
MCV	Known	*KIT* (intergenic)	rs218265	chr4: 55408999	T	1.07	0.67	-0.58 (0.08)	8.9x10^-13^	0.75	0.66	0.65	0.73	0.85
	Known	*CCND3* (intronic)	rs4714548	chr6: 41983431	A	1.02	0.18	-0.58 (0.10)	1.4x10^-9^	0.36	0.16	0.35	0.24	0.13
	Known	*HBS1L / MYB* (intergenic)	rs9389268	chr6: 135419631	A	1.00	0.83	-0.58 (0.10)	3.0x10^-9^	0.78	0.84	0.76	0.89	0.74
	Known	*CITED2* (intergenic)	rs607203	chr6: 139841653	T	1.02	0.07	0.94 (0.15)	1.9x10^-10^	0.24	0.05	0.00	0.02	0.04
	**Novel**	***IDO2* (intergenic)**	**rs141848064**	**chr8: 39876650**	**G**	**1.03**	**0.98**	**-1.41 (0.25)**	**1.1x10**^**-8**^	**0.84**	**0.98**	**1.00**	**1.00**	**1.00**
	Known	*HBB* (missense)	rs334	chr11: 5248232	T	0.86	0.99	3.46 (0.36)	1.1x10^-22^	0.90	>0.99	1.00	1.00	1.00
	Known	*HBA1 / HBA2* (intergenic)	3.8kb del ^d^	chr16: 223447	3.8kb del	NA	0.04	-5.81 (0.18)	2.5x10^-231^	0.16	0.02	0.02	0.02	0.004
	Known	*HBA1 / HBA2* (intergenic)	3.8kb dup ^d^	chr16: 223447	3.8kb dup	NA	0.02	-1.42 (0.25)	1.4x10^-08^	NA	NA	NA	NA	NA
	Known	*TMPRSS6* (missense)	rs855791	chr22: 37462936	A	1.03	0.44	-0.64 (0.07)	1.6x10^-17^	0.10	0.51	0.57	0.54	0.39
	Known^e^	*G6PD* (missense)	rs1050828	chrX: 153764217	C	1.04	0.98	-1.92 (0.22)	1.30x10^-17^	0.87	0.99	1.00	1.00	1.00

Bolding denotes novel associations.

^a^ indicates previous association with other RBC traits, but not with RDW.

^b^ previously reported low-frequency allele (MAF<0.01) observed as significant in this study.

^c^ Allele frequencies provided from HaploReg v4.1 as frequencies not reported in 1000 Genomes.

^d^ The re-typed structural variant calls determined using Genvisis software.

^e^ Analysis on the X chromosome included X chromosome-based eigenvectors and relatedness matrix (sex-stratified results presented in S10 Table).

1000 Genomes superpopulations: AFR = African, AMR = American continents, EUR = European, EAS = East Asian, and SAS = South Asian. CA, coded allele; CAF, coded allele frequency; CNV, copy number variant; SE, standard error; HCT, hematocrit; HGB, hemoglobin; MCH, mean corpuscular hemoglobin; MCHC, mean corpuscular hemoglobin concentration; MCV, mean corpuscular volume; RBC, red blood cell count; RDW, red cell distribution width."oevar" is the imputation quality defined as the ratio of the observed variance of imputed dosage to the expected binomial variance.

### Genomic loci previously known to be associated with RBC traits and generalization to Hispanics/Latinos

Of the 24 genomic regions harboring variants that reach genome-wide significance for association with RBC traits in HCHS/SOL, 17 have been previously found to associate with RBC traits either through GWAS and/or Mendelian RBC disorders. Genomic regions and variants previously implicated in Mendelian RBC disorders include the African ancestral alleles for sickle cell trait/anemia or hemoglobin S (*HBB* rs334); hemoglobin C (*HBB* rs33930165); the common African form of G6PD A- deficiency (rs1050828); the 3.8kb alpha-globin gene deletion responsible for alpha-thalassemia trait (esv2676630); and a proxy SNP (rs2032451) for the European hereditary hemochromatosis (HFE) p.H63D allele.

At 13 of the 17 previously reported RBC loci, the lead variant for the trait detected in HCHS/SOL Hispanics was the same as the previously reported index SNP in European-, African-, or Asian-descent individuals or a strong linkage disequilibrium (LD) proxy (r^2^ >0.8) for the variant, where LD was measured in the relevant ancestral population in 1000 Genomes. There were four cases in which the lead variant in HCHS/SOL was not an LD equivalent to the reported index SNP. The first, rs607203 (MAF = 0.07), is a lead SNP for MCH and MCV association loci located within a DNaseI hypersensitive region on chromosome 6q24 approximately 146kb upstream of *CITED2*. Rs607203 is not in strong LD (HCHS/SOL r^2^ between 0.06 and 0.11) with any of the previously reported *CITED2* European or Japanese index SNPs (rs590856, rs643381, rs628751, rs668459, rs632057), and therefore appears to represent an independent signal in the *CITED2* locus. Among 1000 Genomes super-populations, the frequency of rs607203 is highest in African (AFR) (MAF = 0.14) populations; uncommon in European (EUR), American admixed (AMR), and South Asian (SAS) (MAF<0.05) populations; and monomorphic in East Asian (EAS) populations. A second exception is rs4714548, an intronic SNP of *CCND3* associated with MCV. This HCHS/SOL lead SNP exhibits weak or no LD (HCHS/SOL r^2^<0.1) with any of the *CCND3* index SNPs previously reported in Europeans (rs9349204, rs9349205) or Japanese (rs3218097) populations. Additionally, we report novel associations for two of the variants significantly associated with RDW in HCHS/SOL: *SLC12A7* rs4565255 and *TMPRSS6* rs855791. *SLC12A7* rs4565255 is a proxy for rs4580814, which was previously associated with MCHC in Japanese populations[9]. *TMPRSS6* rs855791 has been previously associated with multiple red cell and iron-related phenotypes, but not with RDW[6, 8, 9].

To formally assess whether variants previously associated with RBC traits in populations of European, Asian, and African ancestry generalized to HCHS/SOL Hispanics/Latinos, we used a directional FDR approach. Of 251 unique published SNP associations with any of the seven RBC traits, 146 (58%) generalized to HCHS/SOL (**S4 Table**). The proportion of loci generalized varied by RBC trait: 5 of 13 HCT variants generalized (38% of SNPs, 42% of loci); 17 of 42 HGB variants generalized (40% of SNPs, 37% of loci); 24 of 33 RBC variants generalized (73% of SNPs, 61% of loci); 38 of 61 MCH variants generalized (62% of SNPs, 61% of loci); 12 of 25 MCHC variants generalized (48% of SNPs, 33% of loci); 49 of 76 MCV variants generalized (64% of SNPs, 58% of loci); and the only variant previously associated with RDW generalized.

### Discovery and replication of new loci associated with RBC traits

The seven remaining genome-wide significant variants in the HCHS/SOL discovery sample were at previously undetected loci (**Table 1**), and three of these variants replicated in a meta-analysis of three independent Hispanic/Latino samples (**Table 2**, **S2 Table**). The replicated loci are (1) chromosome 1q32.3 *PROX1* rs3754140 (MAF = 0.39, replication p = 5.2x10^-3^) associated with HCT; (2) chromosome 5q23.3 *SLC12A2* rs17764730 (MAF = 0.18, replication p = 1.6x10^-3^) associated with RDW; and (3) chromosome 14q11.2 *PSMB5* rs7147308 (MAF = 0.30, replication p = 1.4x10^-5^) associated with RDW. The four loci that did not meet the Bonferroni-corrected replication threshold (P< 0.0071) are (1) *RBFOX3* rs76539504 associated with RBC count (MAF = 0.04, replication p = 0.31); (2) *MCTP2* rs111473449 (MAF = 0.03, replication p = 0.037); (3) an intergenic variant on chromosome 1q31 (rs6685034, MAF = 0.41, replication p = 0.26) associated with RDW; and (4) *IDO2* rs141848064 (MAF = 0.02, replication p = 0.72) associated with MCV.

**Table 2:** Replication of HCHS/SOL GWAS discovery loci in Hispanic/Latino populations.

Trait	Locus	rsID		Discovery		MESA Results		MSSM Results		WHI Results		Replication Meta-analysis	
			Coded Allele	Beta (SE)	p-value	Beta (SE)	p-value	Beta (SE)	p-value	Beta (SE)	p-value	Beta (SE)	p-value
**HCT**	*PROX1*	rs3754140	T	-0.24 (0.05)	5.7x10^-8^	-0.27 (0.18)	0.14	-0.49 (0.28)	0.08	-0.148 (0.072)	0.048	-0.18 (0.07)	5.2x10^-3^
**RBC**	*RBFOX3*	rs76539504	T	0.07 (0.01)	1.4x10^-8^	-0.163 (0.066)	0.16	0.062 (0.051)	0.23	-0.044 (0.048)	0.36	-0.03 (0.03)	0.31
**RDW**	Chr 1q31	rs6685034	A	-0.02 (0.003)	4.8x10^-8^	0.019 (0.012)	0.14	0.004 (0.067)	0.96	-0.006 (0.018)	0.73	0.011 (0.010)	0.26
**RDW**	*SLC12A2*	rs17764730	T	-0.01 (0.001)	8.8x10^-13^	-0.007 (0.005)	0.18	-0.049 (0.036)	0.17	-0.012 (0.004)	0.005	-0.011 (0.003)	1.6x10^-3^
**RDW**	*PSMB5*	rs7147308	C	-0.007 (0.001)	5.8x10^-9^	-0.010 (0.005)	0.04	-0.031 (0.027)	0.25	-0.014 (0.004)	2x10^-4^	-0.013 (0.003)	1.4x10^-5^
**RDW**	*MCTP2*	rs111473449	G	-0.02 (0.003)	3.2x10^-8^	-0.033 (0.011)	0.004	0.102 (0.056)	0.07	-0.004 (0.012)	0.76	-0.017 (0.008)	0.037
**MCV**	*IDO2*	rs141848064	T	1.41 (0.25)	1.1x10^-8^	0.336 (1.02)	0.74	N/A	N/A	-0.742 (0.935)	0.43	-0.248 (0.688)	0.72

MESA: Multiethnic Study of Atherosclerosis, n = 781 to 784; MSSM: Icahn Mt. Sinai School of Medicine, n = 2,621 to 2,785; WHI: Women’s Health Initiative, n = 1,205 or 3,537 (rs3754140 only). N/A: not applicable.

### Functional analysis of new loci associated with RBC traits

At each of the three replicated discovery RBC-associated loci, we evaluated the functional genomic annotation and regulatory potential of the lead variant and any proxy variants (r^2^≥0.8 in HCHS/SOL) in erythroid cells to determine the most likely causal variant(s). We identified the following variants as the most likely functional candidates: three intronic SNPs of *PROX1* (rs7541039, rs7517701, and rs4282786) located within the same erythroid enhancer; one SNP 3’ of *PSMB5* (rs11846575); and rs3812049, which is located in a bi-directional promoter between *SLC12A2* and an anti-sense long noncoding RNA, *LINC01184* (**S5 and S6 Tables**).

**Fig 1:**
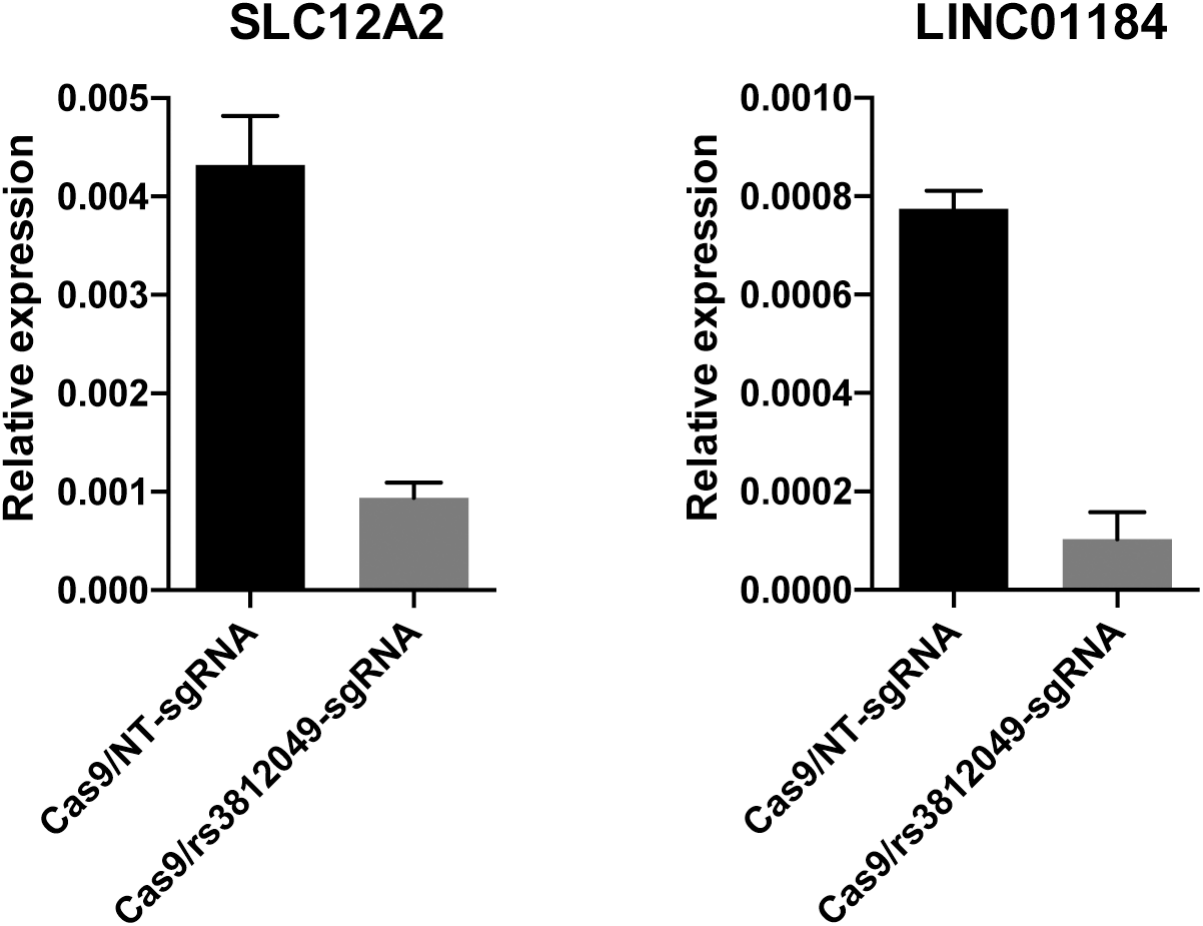
Small indels around rs3812049 reduce expression of both *SLC12A2* and *LINC01184* in HUDEP-2 cells. HUDEP-2 human erythroid precursor cells were transduced with lentivirus expressing Cas9 and a guide RNA, either nontargeting (NT) or targeting cleavage at rs3812049, and selected with antibiotics. Seven days after transduction, expression of *SLC12A2* and *LINC01184* in the population of edited cells was measured by quantitative reverse transcription PCR. Experiment was performed in biologic triplicate. Bars indicate means and error bars indicate standard deviation. T-tests showed significant differences in expression of both *SLC12A* and *LINC01184* upon introduction of indels around rs3812049 (p < 0.01 for each comparison to unedited controls).

We next performed mutagenesis analysis of the regions containing the *PROX1*, *PSMB5*, and *SLC12A2* candidate causal variants using CRISPR-Cas9 genome editing to disrupt the respective putative regulatory elements in human umbilical cord-derived erythroid progenitor (HUDEP-2) cells (oligonucleotide sequences described in **S7 Table**). At the *SLC12A2* locus, a single guide RNA was expressed along with Cas9 to produce indels surrounding the predicted functional SNP rs3812049. These edits resulted in a substantial decrease in expression of both *SLC12A2* and *LINC01184* (**Fig 1**). Differentiation of erythroid cells was not obviously affected by disruption of the bi-directional promoter site. In a separate mutagenesis experiment, deletion of the third exon of *LINC01184* resulted in a 3-fold reduction in *LINC01184* expression, but did not appear to exhibit substantial *cis* effects on *SLC12A2* expression (**S3 Fig**). While the candidate regulatory region of *PROX1* is located within an erythroid enhancer, *PROX1* itself is not expressed in human erythroid cells including HUDEP-2, suggesting that the enhancer element might regulate a distal target. However, a 700 base-pair biallelic deletion of the *PROX1* intronic region containing rs7541039, rs7517701, and rs4282786 did not show any effect on HUDEP-2 cell maturation or on expression of neighboring genes *SMYD2* and *CENPF*, both located within 300 kb of the putative enhancer element (**S4 Fig**). Similarly, deletion of the putative enhancer downstream of *PSMB5* did not significantly alter expression of *PSMB5* or neighboring genes (*PRMT5*, *HAUS4*, *C14ORF93*, and *ACIN1*) that are both expressed in erythroid precursors and located within the same topologically associated domain of K562 cells (**S4 Fig**).

### Additional analysis of the alpha-globin copy number variant

Since the quality of structural variants imputed from 1000 Genomes may be lower than single nucleotide variants, we applied a specialized copy number variant (CNV) calling algorithm to re-type the key 3.8kb alpha-globin structural variant using raw probe intensity data from the custom 2.5M Illumina genotyping array used in HCHS/SOL, as described under **Methods**. Comparison of the CNV genotype calls to those for esv2676630 imputed from 1000 Genomes revealed that genotype calling using imputation appears to result in “under-calling” of the 3.8kb deletion, especially homozygous deletions (**S8 Table**). In addition, there are a number of individuals in HCHS/SOL who carry a 3.8kb duplication (3 or 4 copies of the structural variant), which are mis-called by 1000 Genomes imputation as wild-type. Notably, the improvement in genotype accuracy with the CNV calling algorithm resulted in a nearly two-fold increase in effect size for MCH and MCV (**Table 3**) compared to 1000 Genomes imputation (**S9 Table**). Therefore conditional association analyses were performed using alpha-globin deletion/duplication genotypes derived from the CNV calling algorithm.

**Table 3:** Independent signals at GWAS loci identified by conditional analysis of HCHS/SOL participants.

Trait	Locus	Location	rsID	chr: position	Coded/Alt Allele	CAF	oevar	CR^b^	beta (SE)	p-value	1000 Genomes Allele Frequencies				
											EUR	AFR	AMR	SAS	EAS
MCH	16p13.3	3.8kb deletion^a^	esv2676630	chr16:223447	Deletion/ Reference	0.04	NA	1	-2.60 (0.06)	<2.5x10^-231^	0.004	0.16	0.02	0.02	0.02
		**2.3kb 5' of HBA2**	**rs145546625**	**chr16:220583**	**C/T**	**0.93**	**0.99**	**3**	**0.39 (0.06)**	**2.70x10**^**-12**^	**1.00**	**1.00**	**0.92**	**1.00**	**1.00**
MCV	11p15.4	*HBB* (missense)	rs334	chr11:5248232	T/A	0.99	0.86	1	2.42 (0.37)	3.7x10^-11^	1.00	0.90	0.99	1.00	1.00
		*MMP26-OR51 genes*(intergenic)	rs113342804	chr11:4953240	A/G	0.99	1.01	2	2.28 (0.35)	9.4x10^-11^	1.00	0.96	1.00	1.00	1.00
	16p13.3	3.8kb deletion^a^	esv2676630	chr16:223447	Deletion/ Reference	0.04	NA	1	-5.81 (0.18)	2.5x10^-231^	0.004	0.16	0.02	0.02	0.02
		3.8kb duplication^a^	NA	chr16:223447	Duplication/ Reference	0.02	NA	1	-1.42 (0.25)	1.4x10^-08^	NA	NA	NA	NA	NA
		***HBM* (splice donor)**	**rs148323035**	**chr16:216090**	**T/C**	**0.93**	**0.99**	**3**	**1.07 (0.16)**	**5.60x10**^**-12**^	**1.00**	**1.00**	**0.92**	**1.00**	**1.00**

Rows in bold indicate variants that are Amerindian specific. 1000 Genomes super-populations European (EUR), African (AFR), American (AMR), South Asian (SAS) and East Asian (EAS), were examined to determine global allele frequencies. "oevar" is the imputation quality defined as the ratio of the observed variance of imputed dosage to the expected binomial variance.

^a^ The re-typed structural variant calls determined using Genvisis software.

^b^ CR: during sequential conditional analysis, the round number in which the variant was conditioned for.

### Conditional analysis and identification of secondary, independent association signals

To identify additional independent association signals at known or novel RBC-associated loci, we performed step-wise conditional regression analyses in which we adjusted for the index variant at each genome-wide significant locus. The analysis was repeated with adjustment for each independently associated single variant or structural variant until no further independent signals were identified within that genomic region. Using a significance threshold of α = 5x10^-8^, we identified additional independent variants associated with one or more RBC traits (**Table 3**) in two genomic regions. At the beta-globin locus on chromosome 11p15 containing the index SNP rs334 (sickle cell variant), there was an additional intergenic variant (rs113342804) independently associated with MCV. At the terminal region of chromosome 16p13 containing the alpha-globin locus, we identified two additional low-frequency variants—*HBM*-*HBA2* rs145546625 (or its proxy *HBM* rs148323035 for MCH and MCV) and the 3.8kb alpha-globin duplication (for MCV)—independently of the 3.8kb alpha-globin deletion.

### Admixture mapping analysis

Several variants associated with RBC traits in the HCHS/SOL population are highly differentiated across ancestral populations. The *HBB* rs334, *HBB* rs33930165, esv2676630 alpha-globin 3.8kb gene deletion, and *G6PD* rs1050828 lead variants are derived from an African ancestral background, while the *HFE* hemochromatosis variant rs2032451 (proxy of rs1799945 p.H63D) is common among Europeans and Amerindian populations and much less common among Asians and West Africans. In addition, we note that the two newly reported independent association signals at the chromosome 16 alpha-globin locus—rs148323035/rs145546625 (**Table 3**) and the 3.8kb duplication—appear to be more common among populations of Amerindian ancestry[20, 21]. To assess whether any additional genomic regions might contain ancestrally differentiated SNPs associated with RBC traits, we performed a genome-wide admixture-mapping scan in HCHS/SOL for discovery analysis in each RBC trait. Admixture mapping in HCHS/SOL only detected associations already reported in the initial association testing: the chromosome 11p15 beta-globin region (for MCV); the chromosome 16p13 alpha-globin region (for RBC, HGB, MCV, MCH, MCHC, and RDW); and the RDW association on chromosome 14q11, which corresponds to the *PSMB5* association signal discovered in the HCHS/SOL GWAS (**S5 Fig**). The *PSMB5* index SNP shows large inter-continental allele frequency differences (rs7147308 T allele frequency is 0.87 in AFR, 0.40 in SAS, 0.30 in EUR, 0.21 in AMR, and 0.06 in EAS 1000 Genomes populations).

## Discussion

We performed a GWAS of seven red blood cell traits in a diverse subsample of approximately 12,500 Hispanic/Latino participants of HCHS/SOL from across the continental U.S. We discovered and replicated three genome-wide significant variants (*SLC12A2* rs17764730 and *PSMB5* rs941718 for RDW, and *PROX1* rs3754140 for HCT). We also showed that common African ancestral hemoglobin variants (beta-globin Hb S and Hb C missense variants rs334 and rs33930165, and alpha-globin 3.8kb thalassemia structural variant) and the African G6PD A- variant are associated with variation in RBC traits among the U.S. Hispanic/Latino population. Overall, 58% of previously identified GWAS loci for RBC traits generalized to HCHS/SOL. We additionally provide a more detailed characterization of allelic heterogeneity at the alpha- and beta-globin loci, including a newly identified Amerindian ancestral variant that overlaps a known regulatory region of the alpha-globin gene cluster.

The HCT index SNP rs3754140 is located within a putative enhancer region positioned in the second intron of *PROX1* and is in high LD (r^2^ >0.8) with approximately 30 other intronic *PROX1* variants (**S5 and S6 Tables**). Some of these intronic proxy SNPs (rs7541039, rs7517701, and rs4282786) occur within putative regulatory regions in erythroleukemia or proerythroblast cells, have CADD phred score >10, and therefore represent likely functional candidates. All three of these proxy SNPs are located in a putative enhancer element that exhibits DNaseI hypersensitivity in fetal proerythroblasts and K562 cells. Although enhancers can have distal target genes, a potential target is the enhancer-harboring gene *PROX1*, which has been reported as a negative regulator of hematopoietic stem cell renewal and for which mutations have been found in hematopoietic cell lines and primary blood malignancies[22, 23]. *PROX1* encodes Prospero Homeobox 1, a widely expressed transcription factor involved in the development and differentiation of tissues such as endothelial lymphatic vessels, liver, retina, and pancreas[24]. Several *PROX1* variants (e.g., rs340874, rs340839) located in the 5’ UTR of *PROX1* or adjacent antisense noncoding RNA have been associated with metabolic traits such as fasting glucose, insulin resistance, diabetes, and triglyceride levels[25–27]. The HCT-associated signal we detected in Hispanics/Latino is independent of the previously reported *PROX1* metabolic trait association signal. Molecular analysis, including biallelic deletion of a 700bp region surrounding rs7541039 in the second intron of *PROX1*, showed no effect on transcription of *PROX1*—which does not appear to be expressed in human erythroid precursors—or neighboring genes *SMYD2* and *CENPF*[28]. In light of this information, further investigation of the role of the putative *PROX1* intronic regulatory region and associated genetic variants in hematopoiesis—specifically RBC production—is warranted.

The RDW-associated locus on chromosome 14q11 is located in a gene-rich region. The lead SNP rs941718 and several LD proxies are non-coding variants within or near *PSMB5*, which encodes a 20S core proteasome subunit. From the standpoint of RBC biology, the ubiquitin proteasomal system may be particularly important during erythroid maturation and hemoglobin synthesis to control globin-chain balance and limit potential toxicities of unstable free globin chains[29]. The lead SNP rs941718 is also a blood *cis*-eQTL for nearby genes *HAUS4*, *MRPL52*, *PRMT5-AS1*, and *PRMT5* [30, 31] and has a CADD phred score of 15.8 (**S5 and S6 Tables**). *PRMT5* encodes an arginine methyltransferase involved in binding to the γ-globin promoter and silencing fetal hemoglobin expression, and therefore represents an additional potential mechanism for influencing RBC phenotype[32, 33]. The LD proxy rs11846575, located just 3’ of *PSMB5*, is proximal to a highly tissue-specific erythroid enhancer[34–36] and therefore merits further functional experimentation in the context of erythroid development and hemoglobin synthesis.

The other newly reported RDW-association signal is located on chromosome 5q23 and spans ~100kb including *SLC12A2* and an upstream long non-coding RNA (*LINC01184*) on the antisense strand. *SLC12A2* (which codes for the protein NKCC1) is a sodium-, potassium-, and chloride-ion transporter membrane protein involved in cell-volume regulation and maintenance in kidney, RBC, and other cell types[37]. Genetic variation in other RBC membrane ion-transport proteins (e.g., *PIEZO1*, *SLC4A1*) has been associated with inter-individual variability in RBC traits[13]. The lead SNP at the *SLC12A2* locus (rs17764730) lies within an exon of *LINC01184*. RNA-Seq data indicates that both *SLC12A2* and *LINC01184* are expressed in erythroblasts[35]. The lead SNP is in high LD (r^2^ >0.8) with 23 other variants spanning *SLC12A2* and *LINC01184* (**S5 and S6 Tables**). The strongest functional candidate SNP (rs3812049, imputation quality score 1.006, r^2^ to lead SNP = 0.89) is located within a bi-directional promoter region between the 5' ends of *SLC12A2* and *LINC01184*. Rs3812049 is also positioned within an erythroid DNaseI hypersensitive region and is occupied by multiple transcription factors, including the erythropoietic transcription factors GATA1 and TAL1 in erythroblasts and EGR1 in K562 cells. These observations suggest the possibility that the antisense transcript may be involved in erythrocyte maturation or maintenance by regulating *SLC12A2* in erythrocytes. While this paper was under review, additional variants in the region of SLC12A2 and LINC01184 were reported to be associated with RDW in a predominantly European samples[38, 39].

In human erythroid progenitor cells, we showed that small deletions in the bi-directional promoter region, including directly overlapping the position of rs3812049, lead to reduced expression of both *SLC12A2* and *LINC01184*. Although formally demonstrating the function of the underlying element, these results could be consistent with a model in which rs3812049 alleles differentially modulate promoter activity. While disruption of the bi-directional promoter element did not reveal any differences in erythroid development, *in vitro* conditions may incompletely model a complex trait like RDW that appears highly dependent on appropriate RBC maturation and clearance *in vivo*. Finally, it is interesting to note both the large allele-frequency differences of the *SLC12A2* index variant between African and non-African populations (**Table 1**) and a report of lower erythrocyte NKCC1 protein activity in African Americans compared to whites[40]. This is particularly noteworthy given the established role of NKCC1 in blood pressure regulation, kidney function, and RBC-volume maintenance, and ethnic differences among these traits[37]. Based on our preliminary molecular results, both *SLC12A2* and *LINC01184* should be examined further for their potential roles in erythrocyte and non-erythroid traits.

The HCHS/SOL cohort represents a diverse subsample of Hispanics/Latinos across the U.S., with varying admixture proportions of three continental ancestry groups: Amerindians, Africans, and Europeans. The beta-globin hemoglobin S and hemoglobin C variants, alpha-globin 3.8kb deletion, and G6PD A- variant have previously been shown to contribute to RBC phenotypic variance among U.S. African Americans[41, 42]. Here, we establish that these same common African ancestral hemoglobin and *G6PD* gene variants are associated with quantitative RBC phenotypes among U.S. Hispanics/Latinos. The heterozygous states of each of these inherited RBC conditions are prevalent in populations in Africa, Asia, southern Europe, and South and Central America, and confer a survival advantage against malaria[43]. Even though carriers are generally without clinical sequelae, the heterozygous state of Hb C can induce RBC dehydration, resulting in a higher MCHC[44]. Alpha-globin deletion carriers[1] and sickle cell trait carriers[45] may have lower levels of HCT, MCV, and MCH, and higher RBC counts, due to ineffective erythropoiesis. We also show that the HFE p.H63D variant (rs1799945) is associated with RBC phenotypes in Hispanics. Both C282Y and H63D hemochromatosis mutations are prevalent in Northern Europeans, while H63D appears more broadly in North Africa, the Middle East, and less commonly in Asia. Emigration from Europe over the past 500 years likely introduced C282Y and H63D to Americas and Oceania, leading to a frequency of H63D in Amerindians and Hispanics/Latinos exceeds that of East and South Asians[46, 47].

At the alpha-globin locus, the 3.8kb deletion and duplication generally arise as a result of misalignment of homologous sequences within *HBA1* and *HBA2* and unequal crossing over during recombination. In U.S. Hispanics/Latinos, we observed that the 3.8kb alpha-globin duplication was significantly associated with lower MCV independently of the 3.8kb deletion. This may be due to imbalanced alpha/beta globin-chain synthesis, which may be exacerbated by co-inheritance of other globin gene mutations[48]. Nonetheless, given the caveats of structural variant calling from genotype data, this finding requires additional validation using other molecular techniques. We observed additional allelic heterogeneity at the alpha-globin locus, a novel association signal for MCV and MCH with two Amerindian ancestral variants in high LD (r^2^>0.99): the *HBM* splice-site variant rs148323035, and rs145546625, located ~2 kb upstream of *HBA2*. *HBM* encodes hemoglobin mu, a globin chain similar to the oxygen high-affinity delta-globin found in reptiles and birds that is transcribed in a tightly regulated fashion in erythroid cells, particularly during the terminal differentiation stage[49]. The *HBM* splice donor variant rs148323035 overlaps with a putative regulatory region that spans the transcription start site and first intron of *HBM* and is DNase hypersensitive, occupied by GATA1 and TAL1 in pro-erythroblasts[49].

Overall, generalization analysis revealed that 58% of RBC trait associations identified in GWAS of European-, Asian-, or African-descent populations generalized to HCHS/SOL Hispanics/Latinos. Nearly half of the previously reported genomic regions associated with RBC traits also had at least one variant associated one or more RBC traits in the HCHS/SOL, and 79% of individual SNPs previously reported as significant for more than one RBC trait generalized to HCHS/SOL for at least one of the previously reported traits. These results demonstrate that the same loci are likely involved in RBC trait biology across global populations, whether the functional variants are shared with or differ between ancestral groups. Failure to generalize can occur for one of several reasons, including but not limited to: (1) coverage of the relevant locus on the genotyping array is insufficient for the study population; (2) the originally published variant was a false positive and that locus is not associated with the relevant trait; (3) the power for generalization in HCHS/SOL is low due to the HCHS/SOL study population size; or (4) the power for generalization in HCHS/SOL may be low due to allelic frequency differences between populations.

In summary, we report three novel loci associated with RBC traits in Hispanics/Latinos as well as independent signals within two RBC trait-associated regions previously identified in African descent populations. This includes an Amerindian ancestral variant at the alpha-globin gene cluster that overlaps a known alpha-globin regulatory region. This particular variant is monomorphic among European, Asian, and African ancestral populations. Other Amerindian-specific loci for platelet count or diabetes have been identified among Hispanics/Latinos[50, 51]. These findings emphasize the importance of performing genetic studies in Hispanic/Latino populations.

## Methods

### Study population

The HCHS/SOL is a cohort of 16,415 self-identified Hispanic/Latino persons aged 18–74 years who were selected from households and census block groups in Chicago, IL, Miami, FL, Bronx, NY, and San Diego, CA, as previously described[52]. Study participants self-identified as having Hispanic/Latino background in one of six sub-groups, with the total study population including 6,471 participants identifying as having a Mexican background, 2,728 as Puerto Rican, 2,348 as Cuban, 1,730 as Central American, 1,460 as Dominican, and 1,068 as South American. Individuals were recruited to HCHS/SOL between 2008 and 2011, and underwent a baseline clinical exam that included clinical, lifestyle, and sociodemographic assessment[53]. Based on kinship coefficient among the genotyped individuals, the HCHS/SOL sample includes 204 parent-offspring trios, 1,042 parent-offspring duos, 699 full-sibling pairs, and numerous second- and third-degree relatives. The IRB committees for the HCHS Coordinating Center at UNC Chapel Hill, San Diego State University, University of Illinois at Chicago, University of Miami, and Yeshiva University-Albert Einstein College of Medicine have all reviewed and approved the informed consent documents and study protocol. Written and signed informed consents in the language preferred by the participants are administered and archived at each of the participating field centers. All participants in this publication from HCHS/SOL have consented to use of their genetic and non-genetic data. Anyone not providing consent has been excluded from this analysis. Demographic characteristics and RBC trait descriptive statistics for included study populations are presented in **S2 Table**.

### Red blood cell trait measurement

Whole blood (approximately 58 to 76ml) was collected at Visit 1 for all consenting HCHS/SOL participants by certified technicians trained at their respective field-center institutions. Supplies and procedures were standardized across all field centers; 4ml of whole blood for complete blood count (hemogram) was collected in a tube containing EDTA as an anticoagulant. CBC values were measured from whole blood using an automated hematology analyzer (Sysmex XE-2100, Sysmex America, Inc., Mundelein, IL 60060) at the central laboratory at the University of Minnesota Medical Center, Fairview, in Minneapolis.

### Exclusion criteria

Of the 16,415 individuals in the HCHS/SOL cohort study, 12,803 consented to genotyping and passed QC. Several individuals from the genotyped subset were excluded from the analysis, including individuals with predominantly Asian ancestry (n = 19), pregnant women (n = 8), participants with >5% immature granulocytes (n = 2), end-stage kidney disease (n = 46), hematologic cancer (n = 28), or those undergoing cancer chemotherapy (n = 54). After exclusions, a total 12,502 participants were included for HCT, HGB, RBC, MCH, and MCV; 12,501 for RDW; and 12,500 for MCHC.

### Genotype data cleaning and QC

HCHS/SOL subjects who consented to genetic studies had DNA extracted from whole blood, which was genotyped on the Illumina SOL HCHS Custom 15041502 B3 array. This array comprised the Illumina Omni 2.5M array (HumanOmni2.5-8v1-1) and additional custom content[51, 54]. In order to capture more Amerindian variation, the Omni2.5M array was modified by the addition of custom content comprised of ~150K SNPs selected from the CLM, MXL, and PUR 1000 Genomes Phase I samples for higher informativeness to identify Amerindian continental ancestry and for higher frequency in Amerindian genomic segments. Standard quality assurance/quality control (QA/QC) methods for SNP- and sample-level quality were applied. Quality metrics used to filter SNPs included Illumina/LA Biomed assay-failure indicator, missing call rate (>2%), deviation from Hardy-Weinberg equilibrium (p<10^−5^), Mendelian errors (>3 in 1343 trios or duos), and duplicate sample discordance (>2 in 291 sample pairs). Following genotyping QA/QC procedures, there were 12,803 unique study participants and 2,232,944 SNPs available for imputation.

### Imputation

For imputation, we used 1000 Genomes Project phase 1 reference panel and IMPUTE2 software. Genotypes were initially pre-phased using SHAPEIT2 (v2.r644, www.shapeit.fr), and subsequently imputed using IMPUTE2 software (v2.3.0, https://mathgen.stats.ox.ac.uk/impute/impute_v2.html, last accessed Dec 2016)[54]. Only variants with at least two copies of the minor allele present in any of the four 1000 Genomes continental panels were imputed, yielding a total of 25,568,744 imputed variants (SNPs and indels). Imputed genotype dosages were modeled on a continuous scale from 0 to 2 in order to account for genotype uncertainty. Oevar is an imputation quality metric, defined as the ratio of the observed variance of imputed dosage to the expected binomial variance. Variants with an oevar <0.3 were considered low quality and excluded from analysis. Additional information about imputation and quality metrics is found in Conomos, et al[55].

### Copy number variant genotyping and association analysis at the alpha-globin locus

The SOL Illumina Omni 2.5M array contains five variants (rs2362744, rs4021971, rs4021965, rs11639532, rs2858942) within the 3,811bp alpha-globin structural variant that can be used for determining copy number. Raw probe intensity data (normalized X and Y values) were exported from GenomeStudio as FinalReport files and then imported into the Genvisis software package (http://genvisis.org, last accessed Jan 2017) in order to use its specialized CNV calling algorithm. The first step in the process is to re-compute the Log R ratios (LRRs) using centroids derived from only high-quality samples (standard deviation of the autosomal LRRs <0.32 and genotype call rate >98%). LRRs from a set of ~50,000 curated markers were included in a principal components analysis (PCA) to capture DNA quality, DNA quantity, and batch effects. After regressing out 60 PCs from the raw intensity data, we recomputed LRRs and determined the median LRR value for the five markers in the alpha-globin region. Copy-number (0, 1, 2, 3, or 4) calling for the structural variant was then performed after visual inspection of the cluster boundaries with median LRR on the x-axis and median absolute difference on the y-axis. For RBC phenotype association analyses, genotypes were then coded and analyzed separately for the presence of the 3.8kb alpha-globin deletion (0, 1, or 2 copies) and the presence of the 3.8kb alpha-globin duplication (0, 1, or 2 copies).

### Replication samples

For replication of discovery associations in HCHS/SOL, 1000 Genomes Project phase 1-imputed GWAS data were utilized from three Hispanic/Latino study populations. These included the Women's Health Initiative (WHI) SNP Health Association Resource (SHARe) project (n = 3,454), the Multi-Ethnic Study of Atherosclerosis (MESA) cohort (n = 782), and Mount Sinai Bio*Me* biobank (n = 2,854)[56]. Genotyping in WHI-SHARe and MESA was performed using Affymetrix 6.0 array and imputation was performed with MaCH software[57]. Bio*Me* was genotyped using the Illumina OmniExpressExome beadchip array, phasing was performed using ShapeIt Version 2 release 644 and imputation with Impute version 2.3 using the All 1000 Genomes Project phase 1 integrated variant set (Aug 2012) as the reference.

### Statistical analyses in HCHS/SOL

All outcomes were analyzed using linear mixed-effect models (LMMs), with random effects accounting for inter-individual correlation (due to either relatedness, shared household, or census block group). The covariates (fixed effects) included age, sex, five principal components, recruitment center, current cigarette smoking, sampling weight, and genetic analysis group (Cuban, Dominican, Puerto Rican, Mexican, Central American and South American)[54]. When performing analysis on the X chromosome, we included the first two X chromosome-specific principal components as covariates. Additionally, pairwise genetic relatedness as estimated from the X chromosome was included as a random effect along with the autosomal genetic relatedness matrix. Additionally, since males have only one copy of X chromosome, genotypes on the X chromosome were coded 0, 1, 2 for females and 0, 2 for males. We also conducted three additional analyses for the known G6PD locus on the X chromosome: (1) sex-stratified analysis (**S10 Table)**; (2) genotype-specific analysis in women, since there is evidence for skewed X chromosome-inactivation with age[58] (**S11 Table**); and (3) age-genotype interaction analysis (**S12 Table**).

More information about the principal components, kinship matrix computation, and the genetic analysis groups, is provided in Conomos, et al[54]. Potential inflation was assessed using quantile-quantile plots of the test statistics against the standard normal distribution, and a calculated inflation factor *λ*_*gc*_. We report genome-wide significant results at significance threshold of p-value ≤5.0x10^-8^ and suggestive significance threshold of p-value <1.0x10^-7^ in the HCHS/SOL discovery sample for all variants with MAF = 0.01 and imputation oevar >0.3 All SNPs exceeding genome-wide significance threshold of p-value <1x10^-7^ are described in **S9 Table**.

### Admixture mapping analysis

Local ancestry estimates were previously inferred in the HCHS/SOL[59]. A genome-wide admixture mapping scan was performed using a linear mixed model with covariates and random effects described above, jointly testing the three ancestries (European, African, Amerindian) at each available locus. On the basis of previous simulation results, a nominal p-value of 5.7x10^-5^ yielded a genome-wide type I error of 0.05. There are currently no well-developed, validated methods available for local ancestry estimation on the X chromosome. Hence, we performed admixture mapping analysis only on the autosomes.

### Replication significance criteria

Association testing was performed in each of the three Hispanic replication data sets (WHI, Bio*Me*, MESA) using linear regression and the same RBC trait transformation as the discovery samples, adjusted for age, sex, and principal components. Meta-analysis of results from the 3 independent Hispanic replication study samples was performed using the inverse-variance-weighted method implemented in METAL (http://csg.sph.umich.edu//abecasis/Metal, last accessed Dec 2016). We defined novel, replicated loci as those which exceeded a Bonferroni-corrected significance threshold of p <0.05/7, or 0.0071 (accounting for 7 SNPs carried forward for replication) and are located >1 megabase (Mb) from a previously reported genome-wide significant association signal.

### Conditional analysis

We performed step-wise conditional analysis for each RBC phenotype to identify secondary, independent association signals within 500kb of known and newly discovered GWAS loci. In the first round of the conditional analysis for each trait, we used the same regression model as in the discovery GWAS, with additional adjustment for previously reported or novel variants identified in this study. The list of variants used in conditional analysis of each trait is provided in **S13 Table**. The significance threshold for discovering new, independent association signals was the same as the genome-wide discovery threshold (α = 5.0x10^-8^) as well as MAF≥0.01. Subsequent rounds of conditional analysis were repeated for each genomic region, adding the strongest genome-wide significant variant from the previous round as a covariate in the regression model, until no further genome-wide significant variants satisfying the MAF threshold remained in that region after covariate adjustment. The full models for each trait used in the final round of conditional analysis are listed in **S14 Table**. After obtaining probe intensity-based CNV calls for the 3.8kb alpha-globin CNV, we conducted conditional analysis on chromosome 16 using the calls from the re-typed CNV. The full models for each trait used in these conditional analysis are also listed in **S14 Table.** Conditional analysis with the re-typed 3.8kb alpha-globin CNV was conducted on the subset of 12,390 individuals for whom the re-typed CNV calls were available.

### Generalization analysis

Variants used in generalization analyses were identified by one of two inclusion methods: (1) any variant listed as genome-wide significant for any of the seven RBC traits in our study in the European Bioinformatics Institute GWAS catalog (http://www.ebi.ac.uk, last accessed Jan 2017); or (2) any RBC trait genome-wide-significant variants published in the main text or supplement of an English-language GWAS indexed in PubMed prior to December 2016. (Of note, we did not identify any GWAS published in a language other than English, hence we expect our list of variants identified using these methods to be complete prior to 2017.) We tested each published RBC-associated variant to see whether that association generalized to Hispanics/Latinos. The directional generalization null hypothesis is rejected if there is enough evidence that the published variant is directionally consistent and associated with the outcome in both the discovery study and HCHS/SOL. We evaluated for generalization all available signals previously reported in any GWAS published in English, for all seven traits evaluated in this paper (**S4 Table**). Most of these SNPs were reported in studies of adults of European ancestry, but we also generalized associations from African- and Japanese-ancestry populations. No variants identified in Danjou, 2015, were included in our genotyped or imputed dataset and hence these variants could not be evaluated for generalization[60]. To test the generalization null hypotheses, we computed directional FDR r-values for each of the tested SNPs. Directional r-values were calculated based on one-sided p-values from both the “discovery” study (reported in the literature) and the HCHS/SOL, and based on the number of tests performed in the discovery study, in order to properly account for multiple testing. A SNP was considered generalized if its r-value was <0.05[61]. In generalizing associations reported by Ganesh et al. (2009), we did not employ directional control since Ganesh, et al., (2009) did not report effect sizes or directions[8]. The implication is a slight loss of power.

Generalization analysis was performed by looking up reported SNPs in HCHS/SOL results, in an analysis that mimics the analysis reported in the discovery study. For example, if a trait was reported as an association analysis with the natural-log-transformed trait, we performed the analysis with the same transformation in the HCHS/SOL population. In some cases, as with Kamatani, et al. (2010), we also matched effect-size reporting methods (standard deviations) for ease of comparison. Transformations, when applicable, are described in **S1 Table**. Since the same SNP-trait association may be reported by multiple studies, we counted only unique SNP-trait associations. In instances where more than one study reported associations for the same SNPs and trait, but used different trait transformations, we selected the results from the generalization analysis in which the trait transformation matched our primary analysis.

Since some SNPs are associated with more than one RBC trait, and some genomic regions contain multiple SNPs associated with multiple traits, we summarize the generalization results as follows. Overall, we summarize the number of generalized unique trait-SNP associations (the same SNP may be counted more than once, if associated with more than one trait). Then, for each trait, we summarized (1) the number of unique SNPs, and (2) the number of unique genomic regions. To define genomic regions, we identified specific SNPs, and a 1Mb genomic region around them. Other SNPs within these regions were clumped together. We say that a genomic region generalized for a specific trait if at least one SNP in the region was associated with the trait.

### Functional annotation of novel loci

We assessed any novel, replicated red blood cell associated loci to determine potentially causal variants. At each locus, we determined if the lead or proxy variants (r^2^ ≥ 0.8) were located within putative erythroid regulatory elements, defined on the basis of enrichment for various histone-modification and ChIP-Seq signals in either erythroblasts or the erythroleukemia cell line K562[34–36]. We defined these regulatory regions as follows: enrichment for histone H3K4me1 as an enhancer, enrichment for histone H3K4me3 as a promoter. Variants located within a putative promoter or enhancer, and that overlapped a DNaseI hypersensitive site in proerythroblasts or K562 cells, were prioritized as putatively functional [34, 36, 62]. Regulatory elements often are bound by transcription factors and hence we report ChIP-Seq peak overlaps of key erythroid transcription factors (GATA1, TAL1), and others in proerythroblasts and K562 cells to provide further support for the functional role of putative regulatory elements in erythroid cells[34, 36, 62]. The ENCODE and BLUEPRINT datasets were accessed through the ENCODE analysis Hub and Blueprint Hub respectively via the UCSC genome browser[63, 64]. Datasets from Xu, et al, were accessed from codex (http://codex.stemcells.cam.ac.uk, last accessed Dec 2016)[62, 65]. To hypothesize likely mode of action via which the causal variants influence the trait, we report eQTL targets and or motifs disrupted by prioritized variants using HaploReg v4.1[66]. All the datasets used for functional annotation were mapped to Human GRCh37/hg19 assembly. Functional annotation is summarized in **S5 Table**. We also used *in silico* prediction algorithms to annotate variants. These included RegulomeDB, the Combined Annotation Dependent Depletion (CADD) phred score, GWAVA, and deltaSVM[67–70]. These annotations are summarized in **S6 Table**.

### In vitro analysis of functional candidates within SLC12A2-LINC01184, PSMB5, and PROX1

The CRISPR/Cas9 system was used to mutagenize individual variants or small regions of interest identified during discovery analysis and subsequent bioinformatics interrogation. All oligonucleotide sequences used in CRISPR-Cas9 genome editing experiments are listed in **S7 Table.** The human umbilical cord blood derived erythroid progenitor cell line #2 (HUDEP-2) was cultured and used for genome editing as previously described[71]. Individual and tandem pairs of single chimeric guide RNAs were cloned to lentiviral expression vectors (lentiGuide-Puro, Addgene plasmid 52963). Cells were transduced and selected for lentiviral integrants by antibiotic selection (10 μg/ml blasticidin for lentiCas9-Blast [Addgene plasmid 52962], 1 μg/ml puromycin for lentiGuide-Puro). For *SLC12A2* individual sgRNA promoter editing, indel frequencies were assessed after 7 days by nested PCR followed by amplicon deep sequencing. For *SLC12A2-LINC01184*, *PSMB5*, and *PROX1* interstitial deletions, cells were plated at limiting dilution to isolate clones 7 days after transduction with tandem sgRNAs. Clones with biallelic deletions were characterized by presence of gap PCR amplification with primers outside the deleted segment and absence of PCR amplification from inside the deleted segment. Expression of mRNA of genes of interest was compared to GAPDH expression using quantitative reverse transcription PCR (RT-qPCR) in control and edited HUDEP-2 cells. For *SLC12A2* individual sgRNA promoter editing, the total population of edited cells was evaluated in bulk by RT-qPCR. For *SLC12A2-LINC01184*, *PSMB5*, and *PROX1* interstitial deletions, clones were first identified by PCR screening and then evaluated by RT-qPCR. For differentiation experiments, control and edited HUDEP-2 cells were cultured separately for 4 days in Erythroid Differentiation Media (EDM) with Iscove’s Modified Dulbecco’s Medium (IMDM) (Life Technologies) supplemented with 330 mg/ml holo-transferrin (Sigma), 10 mg/ml recombinant human insulin (Sigma), 2 IU/ml heparin (Sigma), 5% human solvent detergent pooled plasma AB (Rhode Island Blood Center), 3 IU/ml erythropoietin, 100 ng/ml human SCF, (R&D), 1 mg/ml doxycycline, 1% L-glutamine, and 2% penicillin/streptomycin. Subsequently the cells were cultured an additional 4 days in EDM lacking SCF, and then an additional 4 days in EDM lacking both SCF and doxycycline. Erythroid maturation was evaluated by flow cytometry staining with CD71 (eBiosciences), CD235a (eBiosciences), CD49f (Miltenyi), and DRAQ5 (eBiosciences) as well as morphology by May-Grunwald-Giemsa staining, Student's t-tests were used for statistical analysis of results.

### Data availability statement

Genotype data and GWAS results of discovery analysis of all the seven RBC traits can be requested via dbGaP study accession phs000880. Phenotype data can be requested via dbGaP study accession phs000810

## Supplemental data

Supplemental data includes five figures, nine tables, and five Excel spreadsheets.

## Supporting information

S1 Fig Manhattan plots and accompanying QQ plots for seven RBC traits in 12,502 HCHS/SOL Hispanics/Latinos. **A:** Hematocrit; **B:** Hemoglobin; **C:** Red Blood Cell Count; **D:** Red Cell Distribution Width; **E:** Mean Corpuscular Hemoglobin; **F:** Mean Corpuscular Hemoglobin Concentration; **G:** Mean Corpuscular Volume. *All Manhattan plots include only variants with MAF ≥ 0.01. X-axis of Manhattan plots = ordered chromosomes; Y-axis of Manhattan plots = -log10(p-value). X-axis of QQ plots = expected p-value; Y-axis of QQ plots = observed p-value.
(PDF)

S2 Fig Locus-Zoom plots of loci significantly associated with RBC traits. All variants with minor allele count >30 were plotted using Locus-Zoom software and genome build 37/hg19 positions on the x-axis. The left y-axis is the negative log_10_ p-value for the association between each variant and the relevant RBC trait; the gray line represents genome-wide significance (p<5x10^-8^). The left y-axis (blue lines on the plot) is the recombination rate in percent. The lead SNP at each locus is designated with a triangle if the SNP is imputed, and a diamond if the SNP is genotyped. Each symbol represents one variant, with circles for genotyped and x’s for imputed variants. Linkage disequilibrium (correlation, r^2^) with the lead variant in HCHS/SOL is indicated by color, with the colors for each level of LD shown in the upper-right corner of the plot. The genes at each locus are aligned underneath the plot with the corresponding genomic positions.
(PDF)

S3 Fig Impact of deletion of LINC01184 Exon-3 on expression of LINC01184 and SLC12A2. HUDEP-2 human erythroid precursor cells were transduced with lentivirus expressing Cas9 and a pair of guide RNAs targeting cleavages flanking exon-3 of *LINC01184*. After limiting dilution, clones were screened by PCR for deletion of *LINC01184* exon-3. Twelve clones with biallelic deletion of *LINC01184* exon-3 were identified and utilized for quantitative reverse transcription PCR to measure expression of LINC01184 and SLC12A2. Primers for LINC01184 measurement annealed to sequences at exons 1 and 2, i.e., non-deleted sequences. Data is shown for each of 12 biallelic deletion clones performed in technical triplicate. Gene expression is normalized to the level of parental cells. Lines indicate means and standard deviations.
(DOCX)

S4 Fig Small indels in DNase I hypersensitive sites do not exhibit cis effects on expression of *PROX1* and *PSMB5* in HUDEP-2 cells. Deletions of DNase I hypersensitive sites (DHSs) at *PSMB5* and *PROX1* loci were not associated with significant gene expression changes in cis. HUDEP-2 human erythroid precursor cells were transduced with lentivirus expressing Cas9 and a pair of guide RNAs targeting cleavages flanking DHSs at *PSMB5* and *PROX1*. After limiting dilution, clones were screened by PCR for deletion of DHSs. Biallelic deletion clones were identified and utilized for quantitative reverse transcription PCR to measure expression of neighboring genes. As a control, nondeletion clones were isolated in parallel. Data is shown for RT-qPCR for indicated gene in a single clone, normalized to GAPDH, and then to median of the nondeletion clones. Each measurement was performed in technical triplicate. Lines indicate medians of each set of clones. No significant differences were identified between deletion and nondeletion clones (p > 0.05 for all comparisons).
(TIF)

S5 Fig Manhattan plots from admixture mapping analysis of RBC traits in HCHS/SOL participants. X-axis of Manhattan plots = ordered autosomal chromosomes; Y-axis of Manhattan plots = -log10(p-value). The X chromosome was not evaluated because established methods for admixture mapping of this chromosome are not available.
(DOCX)

S1 Table Red blood cell trait descriptions. Genomic inflation factor refers to the ratio between the median test statistics value and the expected median for variants with MAF ≥ 0.01.
(DOCX)

S2 Table Characteristics of discovery and replication cohorts. *Units for each trait are as follows: Hematocrit, %; Hemoglobin, g/dL; RBC count, cells x10^9^; RDW, %; MCH, pg; MCHC, g/dL; MCV, fL. Population means for hematocrit and hemoglobin are presented as sex-stratified due to significant differences between adult males and females.
** Hematocrit and hemoglobin were available at the baseline exam for WHI SHARe in 3,539 participants. The remaining measures were available in a sub-sample of 1,205 WHI SHARe participants.
(DOCX)

S3 Table Association results in the six genetic subgroups for genetic variants significantly associated with red blood cell traits in HCHS/SOL Hispanics/Latinos. # The full DNA sequence of the deletion for esv2676630 can be found in S9 Table. *het pval: p-value for test of heterogeneity. C/A = coded and alternate alleles. CAF = coded allele frequency. Chromosomal positions refer to hg build19/GRCh37. Sub-groups were generated using self-identified background and genetic principal components analysis.
(XLSX)

S4 Table Generalization of variants previously associated with seven red blood cell traits in European-, Asian-, and African-ancestry populations to HCHS/SOL Hispanics/Latinos. A: Hematocrit; B: Hemoglobin; C: Red Blood Cell Count; D: Red Cell Distribution Width; E: Mean Corpuscular Hemoglobin; F: Mean Corpuscular Hemoglobin Concentration; G: Mean Corpuscular Volume.
CAF = Effect Allele Frequency; N = number of study participants; NR = not reported; "—" = alternate allele not reported. Generalization was based on statistical significance (r ≤ 0.05) and directional consistency with the published variant in HCHS/SOL Hispanics/Latinos.
(XLSX)

S5 Table Summary of findings from the functional annotation of novel red blood cell trait-associated variants and their LD partners (r^2^ ≥0.8) identified in HCHS/SOL. C/A = coded and alternate alleles. CAF = coded allele frequency.
(DOCX)

S6 Table Summary of *in silico* functional prediction algorithm results for novel significant variants and their LD partners (r2 ≥0.8) in discovery and conditional analyses. * Chromosome and base pair position reported from GRCh37/hg19. ^†^ SNP type: 0 = imputed, 2 = genotyped. ^1^ CADD score = PHRED-scale score indicating deleteriousness of variants and all other substitutions in the genome; ^2^ Unmatched score presented from GWAVA; ^3^ Regulome DB score is on a scale from 1 to 7, with lower numbers indicating more evidence for the variant being functional; ^4^ deltaSVM score predicts the impact of SNPs on DNaseI sensitivity. "oevar" is defined as the ratio of the observed variance of imputed dosage to the expected binomial variance.
(XLSX)

S7 Table Oligonucleotide sequences used in CRISPR-Cas9 genome editing,PCR screening and RT-qPCR quantification. F = forward, R = reverse. Chromosomal positions refer to hg build19/GRCh37.
(DOCX)

S8 Table Comparison of 1000 genomes phase I and re-typed (based on probe intensity) deletion genotype calls for the alpha globin 3.8kb deletion. * value of 0 = 0 copies of 3.8kb deletion, 1 = 1 copy of deletion, 2 = 2 copies of deletion.
(DOCX)

S9 Table All variants reaching suggestive significance (1E-7) for association with seven red blood cell traits in HCHS/SOL. A: Hematocrit; B: Hemoglobin; C: Red Blood Cell Count; D: Red Cell Distribution Width; E: Mean Corpuscular Hemoglobin; F: Mean Corpuscular Hemoglobin Concentration; G: Mean Corpuscular Volume. Variants with a low imputation value (oevar < 0.3) were not included in association analyses. Variants with minor allele frequency (MAF) < 0.01 excluded. ^†^ imputed calls for esv2676630 were used in these analyses (see Methods).
(XLSX)

S10 Table Sex-stratified results for genome-wide significant X-chromosome associations. Chromosomal positions are aligned to build hg19/GRCh37. Alt = alternative; CAF = coded allele frequency; MCH = mean corpuscular hemoglobin; MCV = mean corpuscular volume; RBC = red blood cell count; RDW = red cell distribution width; SE = standard error.
(DOCX)

S11 Table Genotype-specific association results for lead X chromosome variant in HCHS/SOL female participants. Chromosomal positions are aligned to build hg19/GRCh37. Alt = alternative; CAF = coded allele frequency; MCH = mean corpuscular hemoglobin; MCV = mean corpuscular volume; RBC = red blood cell count; RDW = red cell distribution width; SE = standard error.
(DOCX)

S12 Table Interaction results of age and lead X chromosome variant genotype in HCHS/SOL female participants. Chromosomal positions are aligned to build hg19/GRCh37. Alt = alternative; CAF = coded allele frequency; MCH = mean corpuscular hemoglobin; MCV = mean corpuscular volume; RBC = red blood cell count; RDW = red cell distribution width; SE = standard error.
(DOCX)

S13 Table List of all variants used in conditional analysis. Allele frequencies reported for 1000 Genomes super-populations European (EUR), African (AFR), American (AMR), South Asian (SAS) and East Asian (EAS). HCT, hematocrit; HGB, hemoglobin; MCH, mean corpuscular hemoglobin; MCHC, mean corpuscular hemoglobin concentration; MCV, mean corpuscular volume; RBC, red blood cell count; RDW, red cell distribution width; NA: “not applicable” because this deletion has not been characterized in 1000 Genomes populations; *I/D: coded allele = insertion, alternative allele = deletion; ^#^ During sequential conditional analysis, the round number in which the variant was conditioned for. CAF, coded allele frequency; SE, standard error. ^†^ imputed calls for esv2676630 were used in all conditional analyses (see Methods).
(XLSX)

S14 Table Full models used for conditional analyses. EV = Eigenvector; HCT = hematocrit; HGB = hemoglobin; MCH = mean corpuscular hemoglobin; MCHC = MCH concentration; MCV = mean corpuscular volume; RBC = red blood cell count; RDW = red cell distribution width; hba_cnv_countDel = intensity-based calls for the alpha gene deletion; hba_cnv_countDupl = intensity-based calls for the alpha gene duplication. ^†^ imputed calls for esv2676630 were used for conditional analyses (see Methods). ^††^ probe intensity-based re-typed calls were used for esv2676630 in the chromosome 16 conditional analyses (see Methods).
(DOCX)
