# Supplementary material for: Genome-wide association study of red blood cell traits in Hispanics/Latinos: The Hispanic Community Health Study/Study of Latinos
Source: PLoS Genet. 2017 Apr 28;13(4):e1006760. doi: 10.1371/journal.pgen.1006760 (PMC5428979; doi:10.1371/journal.pgen.1006760)

Figure S1. Manhattan Plots and accompanying QQ Plots for seven RBC Traits in 12,502 HCHS/SOL Hispanic/Latinos.

Fig S1a. Hematocrit

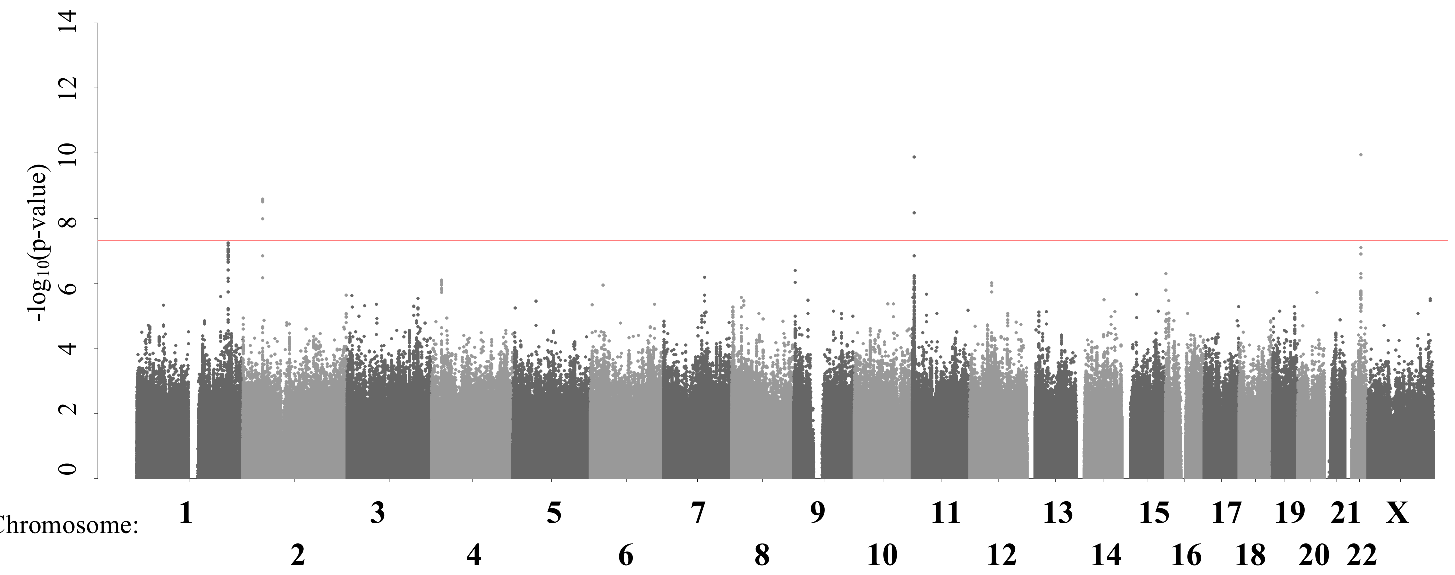

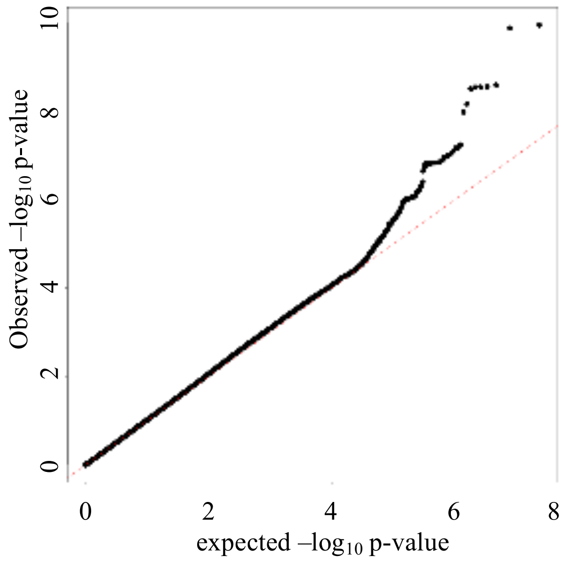

Fig S1b. Hemoglobin

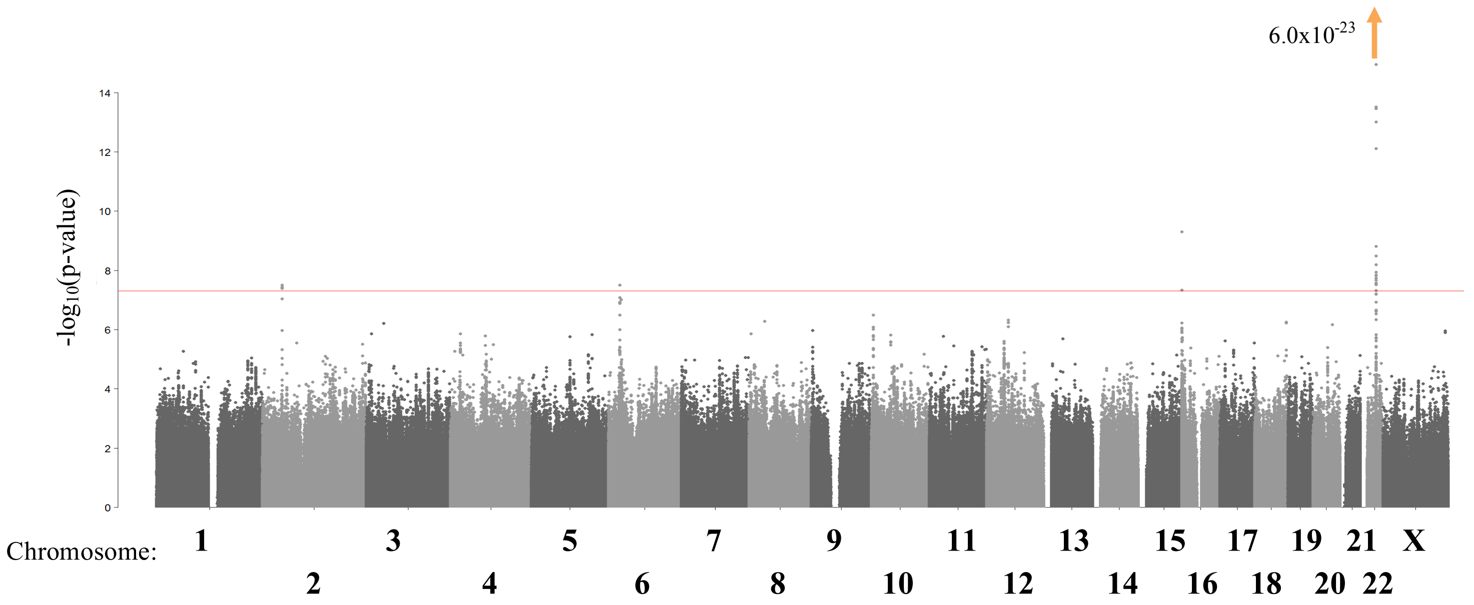

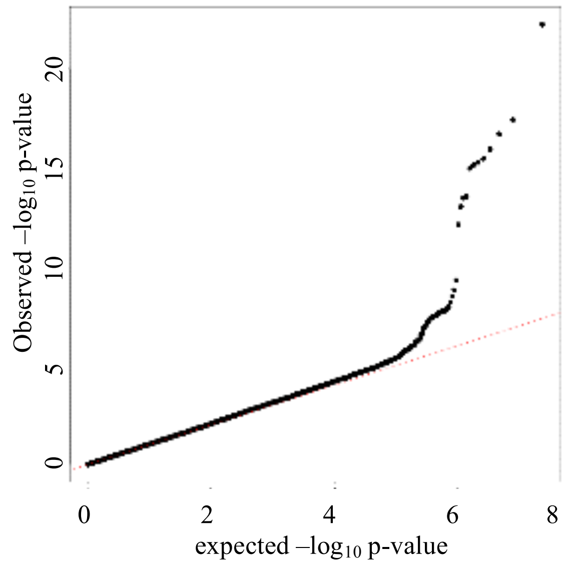

Fig S1c. Red Blood Cell Count

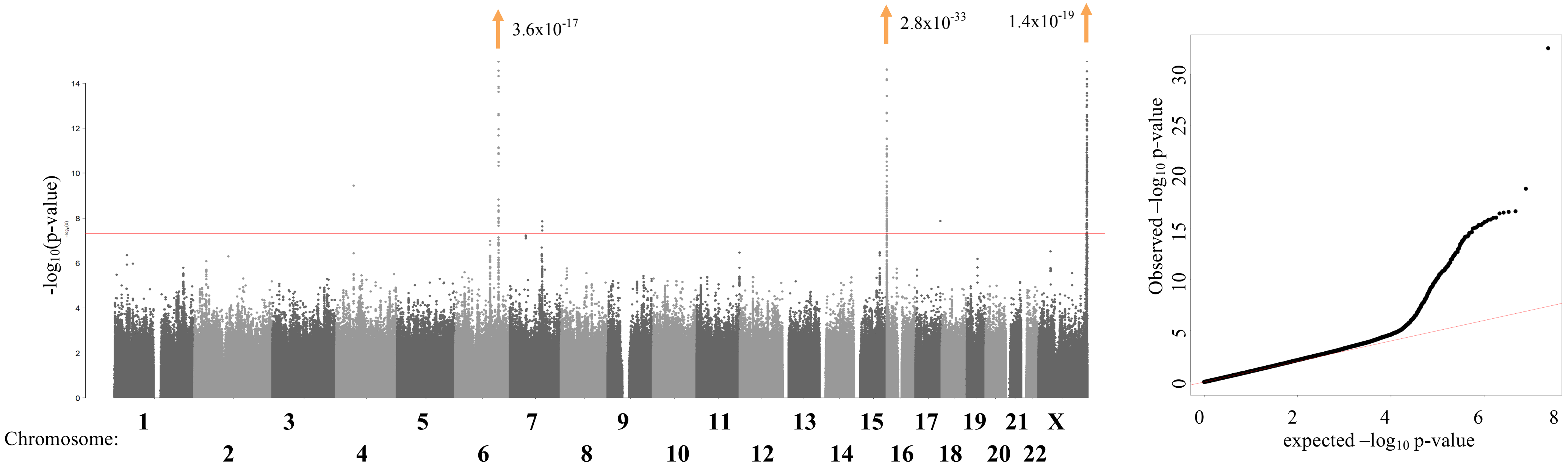

Fig S1d. Red Cell Distribution Width

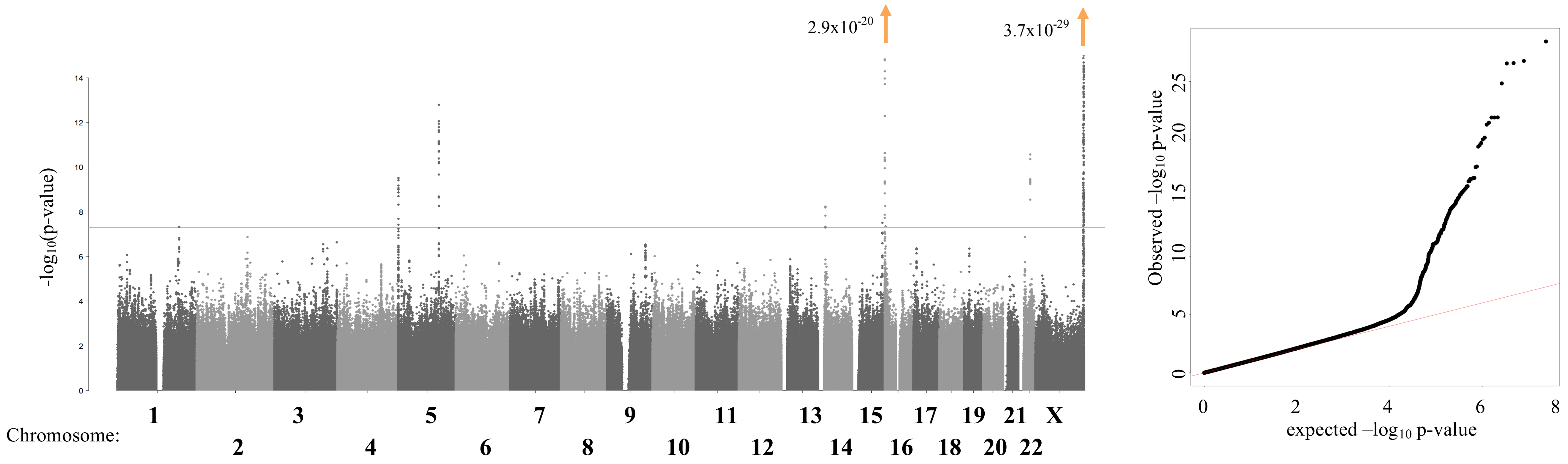

Fig S1e. Mean Corpuscular Hemoglobin

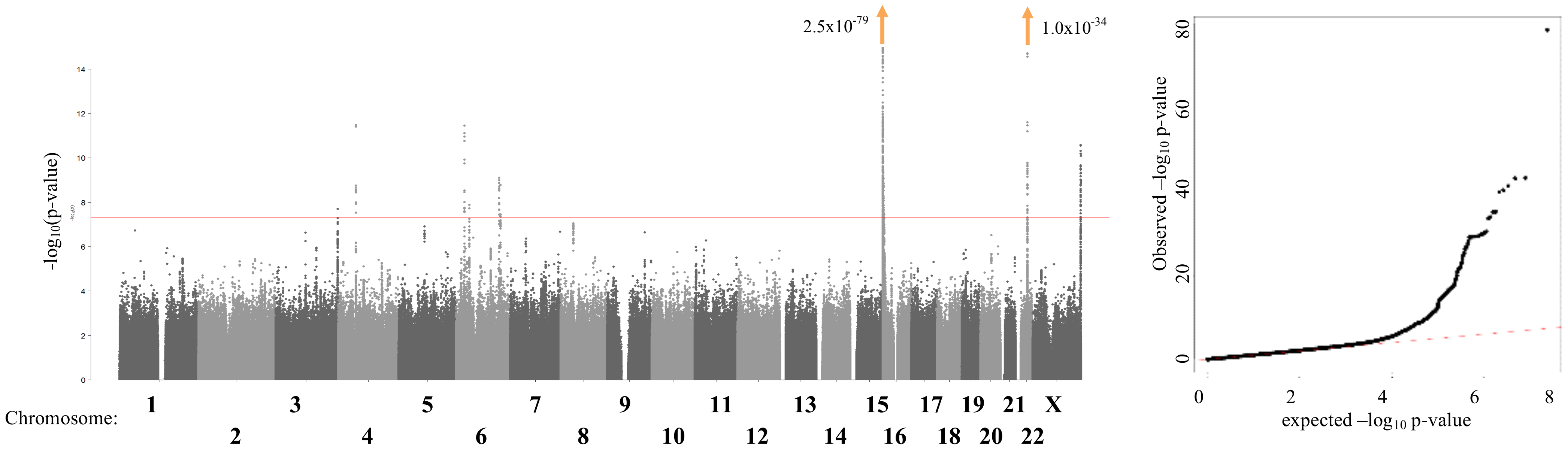

Fig S1f. Mean Corpuscular Hemoglobin Concentration

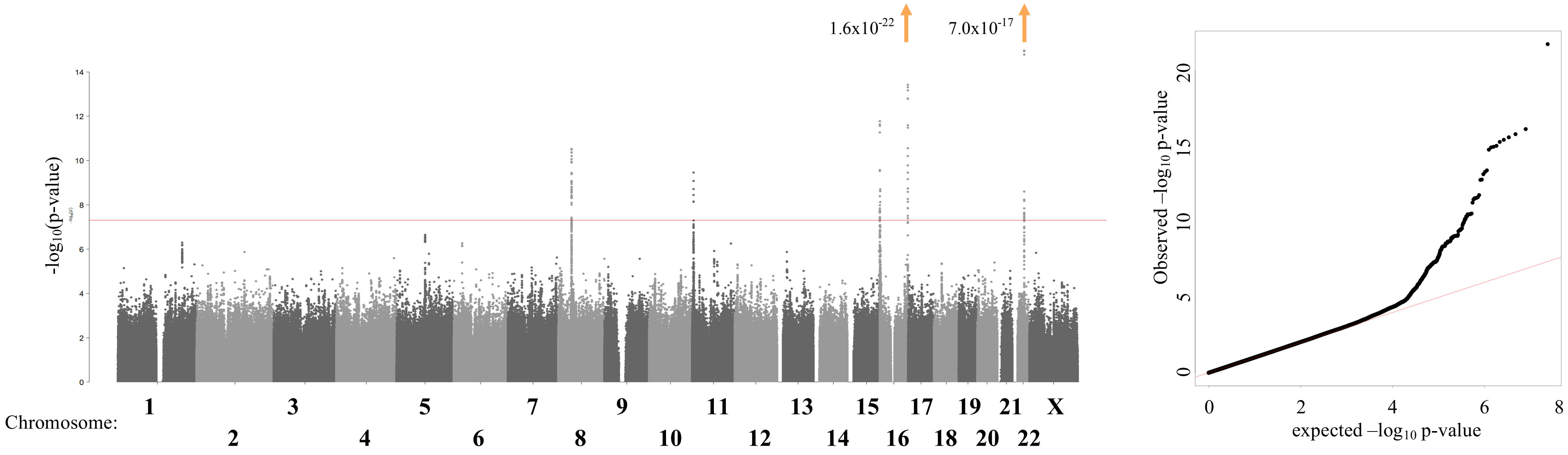

Fig S1g. Mean Corpuscular Volume

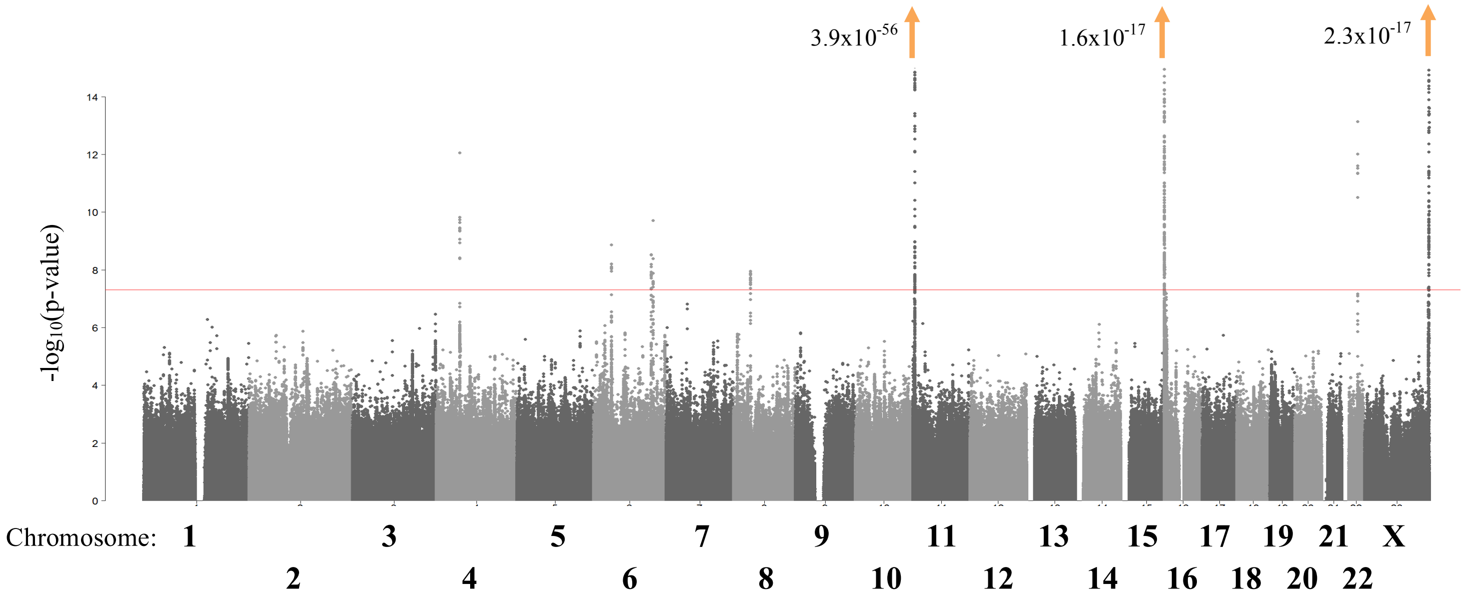

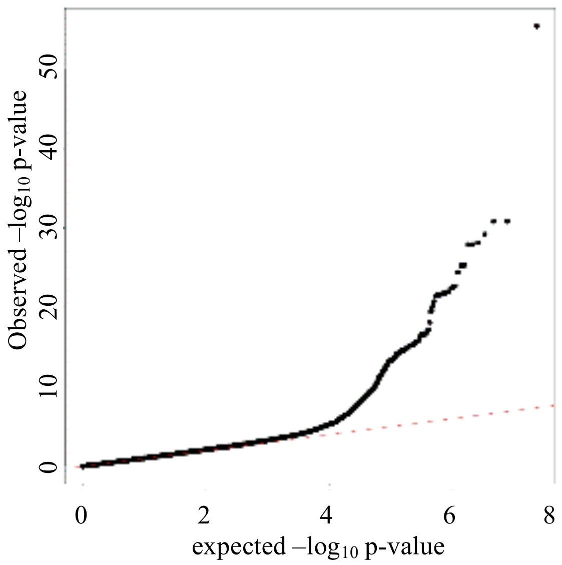

Supplement: S1 Fig — A: Hematocrit; B: Hemoglobin; C: Red Blood Cell Count; D: Red Cell Distribution Width; E: Mean Corpuscular Hemoglobin; F: Mean Corpuscular Hemoglobin Concentration; G: Mean Corpuscular Volume. *All Manhattan plots include only variants with MAF ≥ 0.01. X-axis of Manhattan plots = ordered chromosomes; Y-axis of Manhattan plots = -log10(p-value). X-axis of QQ plots = expected p-value; Y-axis of QQ plots = observed p-value. (PDF) [file pgen.1006760.s001.pdf]
