# Supplementary material for: Genome-wide association study of red blood cell traits in Hispanics/Latinos: The Hispanic Community Health Study/Study of Latinos
Source: PLoS Genet. 2017 Apr 28;13(4):e1006760. doi: 10.1371/journal.pgen.1006760 (PMC5428979; doi:10.1371/journal.pgen.1006760)
Supplement: S2 Fig — All variants with minor allele count >30 were plotted using Locus-Zoom software and genome build 37/hg19 positions on the x-axis. The left y-axis is the negative log10 p-value for the association between each variant and the relevant RBC trait; the gray line represents genome-wide significance (p<5x10^-8^). The left y-axis (blue lines on the plot) is the recombination rate in percent. The lead SNP at each locus is designated with a triangle if the SNP is imputed, and a diamond if the SNP is genotyped. Each symbol represents one variant, with circles for genotyped and x’s for imputed variants. Linkage disequilibrium (correlation, r2) with the lead variant in HCHS/SOL is indicated by color, with the colors for each level of LD shown in the upper-right corner of the plot. The genes at each locus are aligned underneath the plot with the corresponding genomic positions. (PDF) [file pgen.1006760.s002.pdf]

Fig S2 Locus zoom plots for loci significantly associated with red cell traits in HCHS/SOL

Fig S2.1 HCT

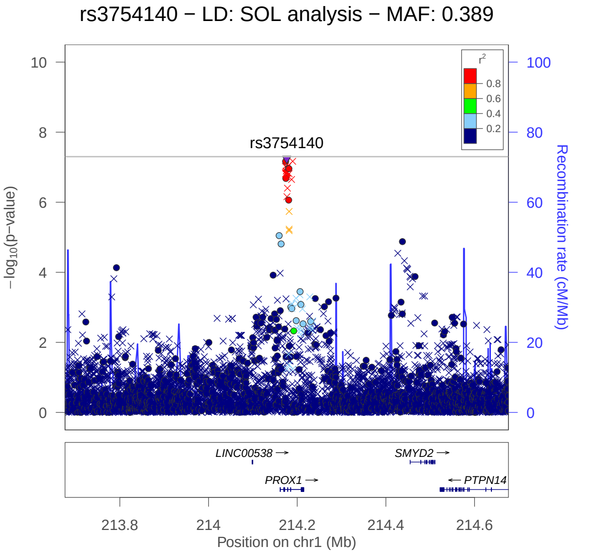

(a) Locus:PROX1, lead variant:rs3754140

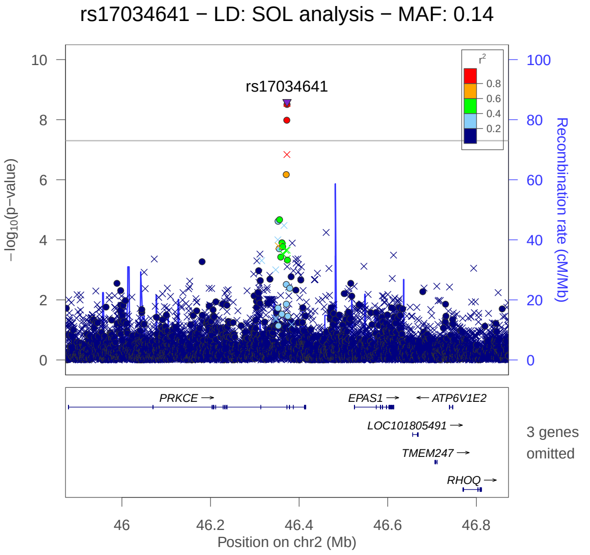

(b) Locus:PRKCE, lead variant:rs17034641

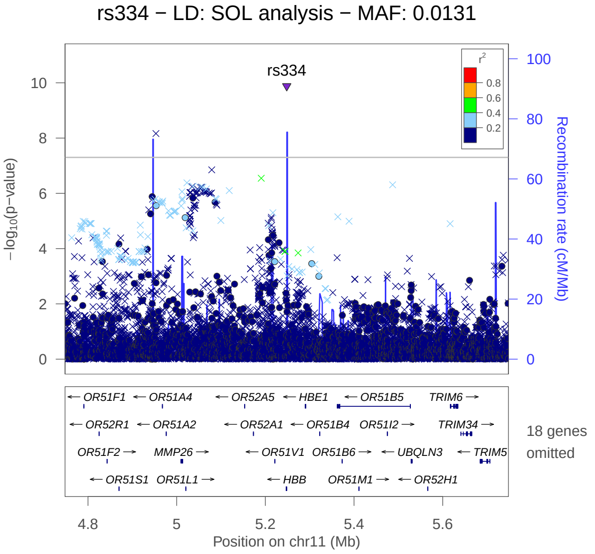

(c) Locus:HBB, lead variant:rs334

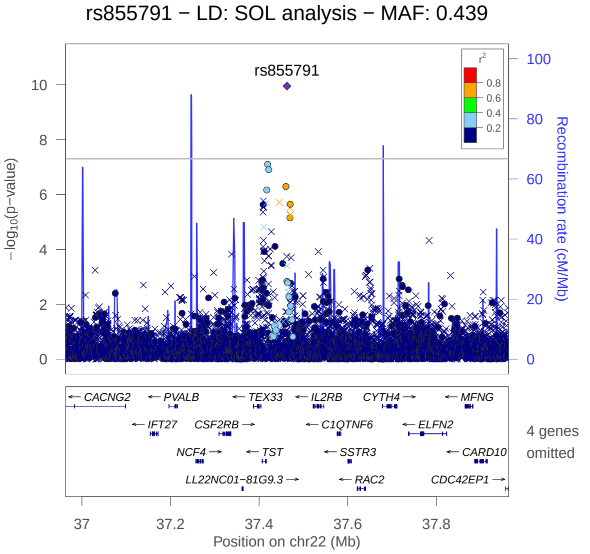

(d) Locus:TMPRSS6, lead variant:rs855791

Fig S2.2 HGB

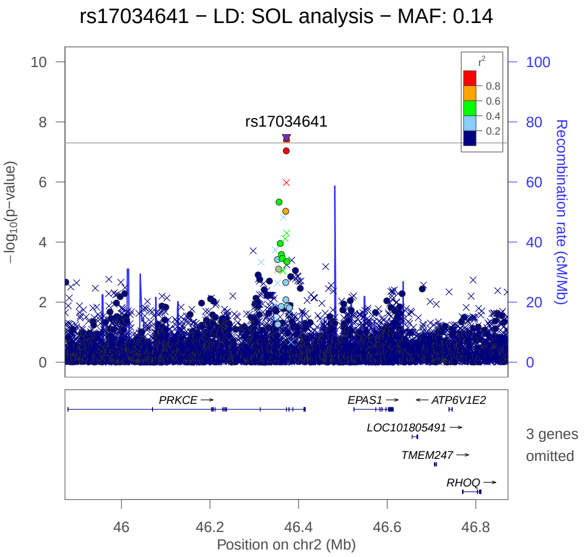

(a) Locus:PRKCE, lead variant:rs17034641

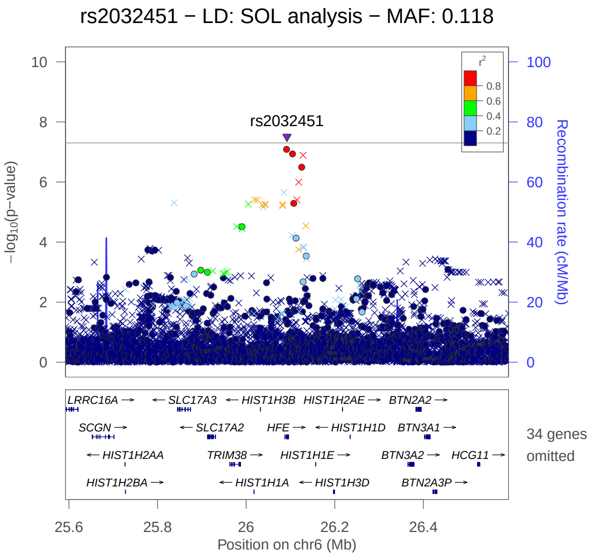

(b) Locus:HFE, lead variant:rs2032451

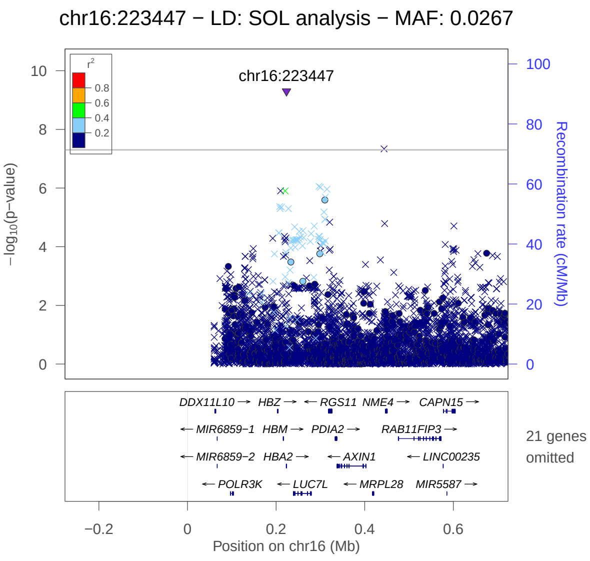

(c) Locus:HBA1/HBA2, lead variant:esv2676630

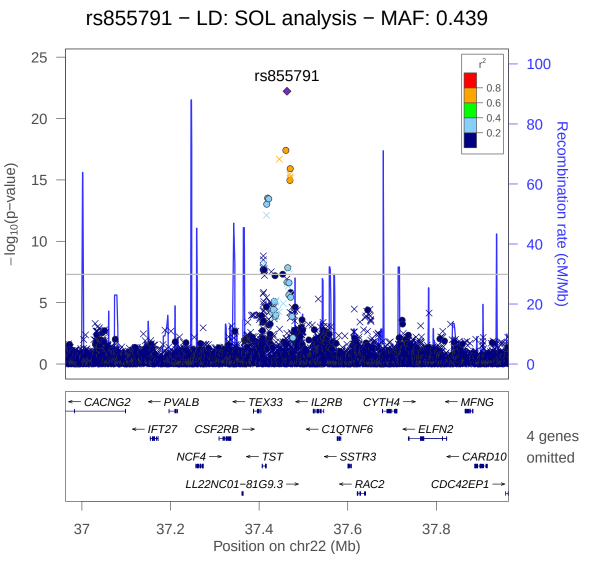

(d) Locus:TMPRSS6, lead variant:rs855791

Fig S2.3 RBC count

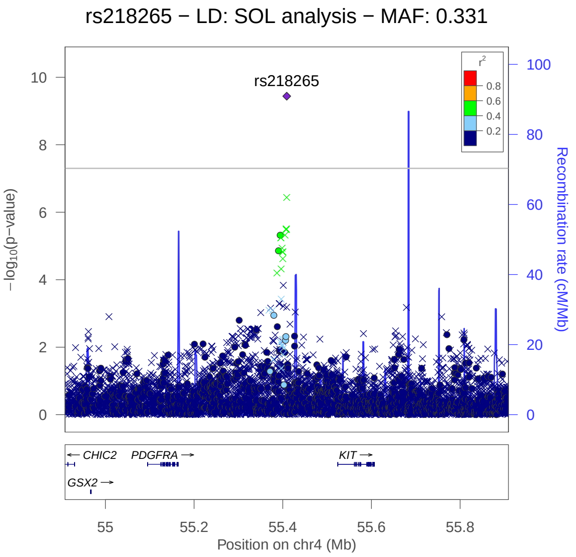

(a) Locus:KIT, lead variant:rs218265

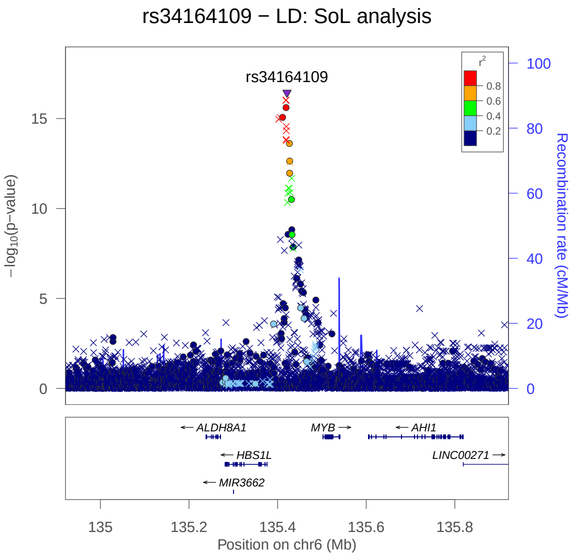

(b) Locus:HBS1L/MYB, lead variant:rs34164109

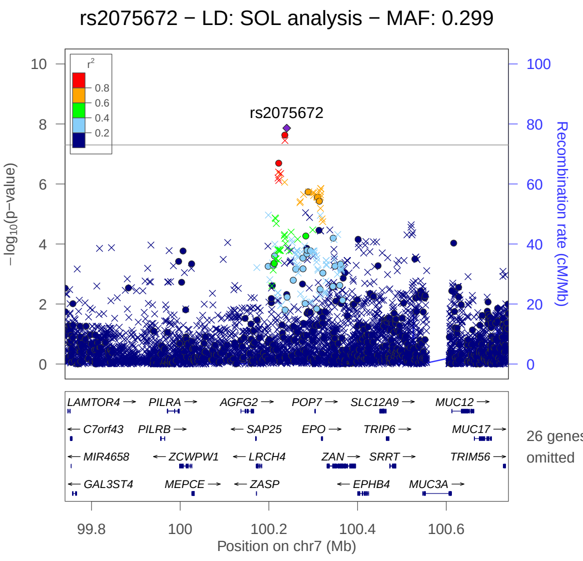

(c) Locus:TFR2, lead variant:rs2075672

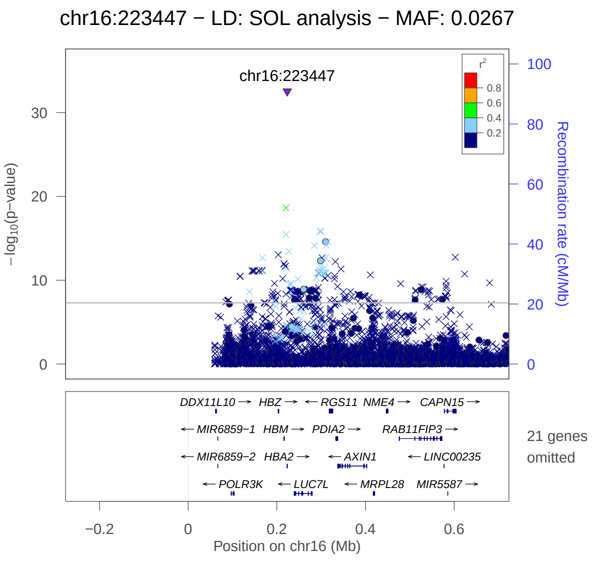

(d) Locus:HBA1/HBA2, lead variant:esv2676630

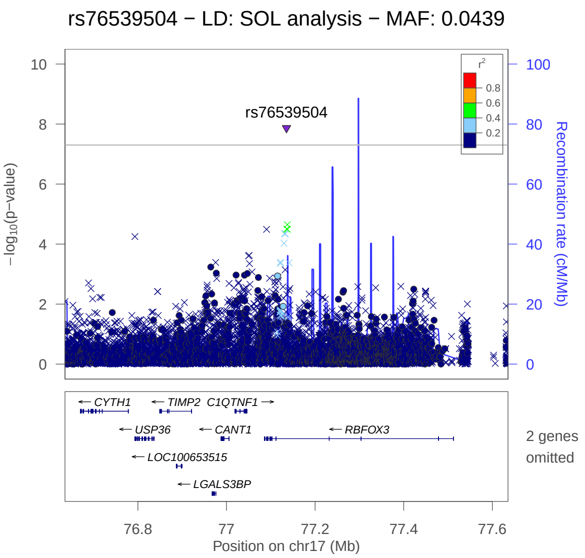

(e) Locus:RBFOX3, lead variant:rs76539504

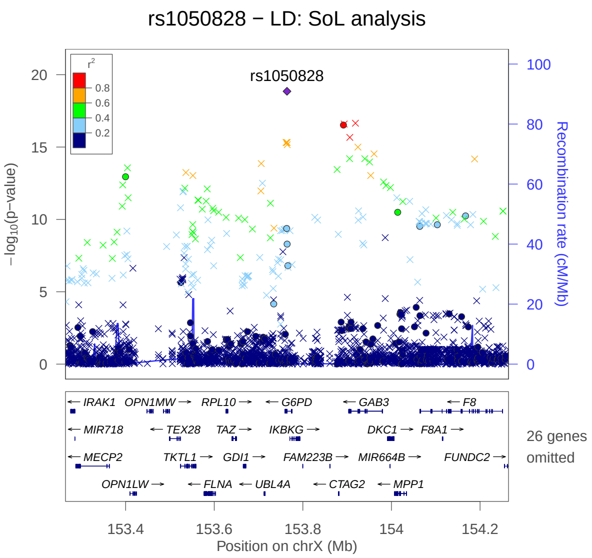

(f) Locus:G6PD, lead variant:rs1050828

Fig S2.4 RDW

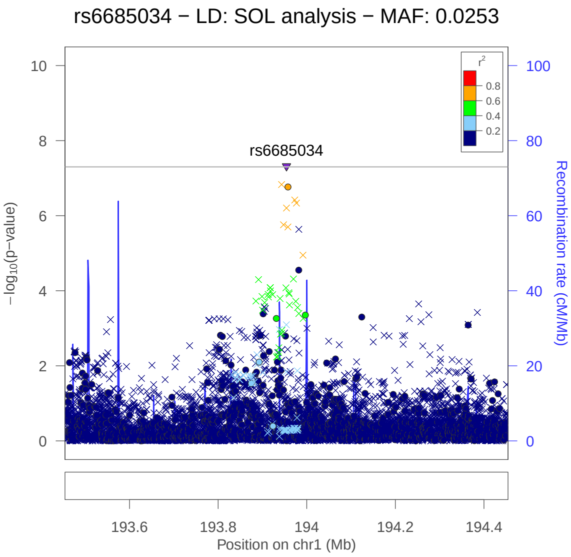

(a) Locus:Intergenic, lead variant:rs6685034

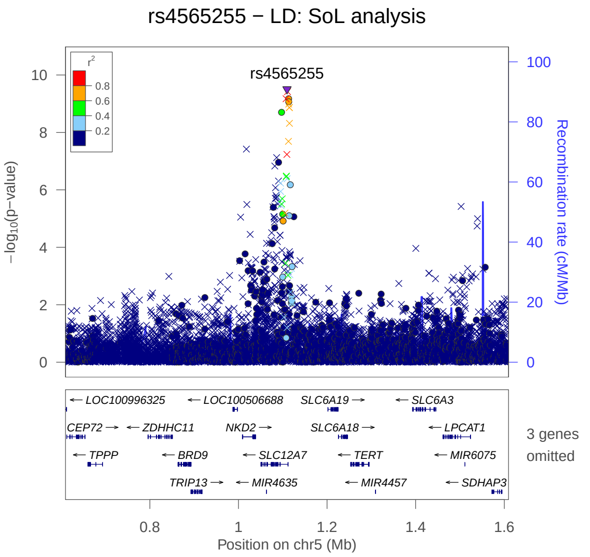

(b) Locus:SLC12A7, lead variant:rs4565255

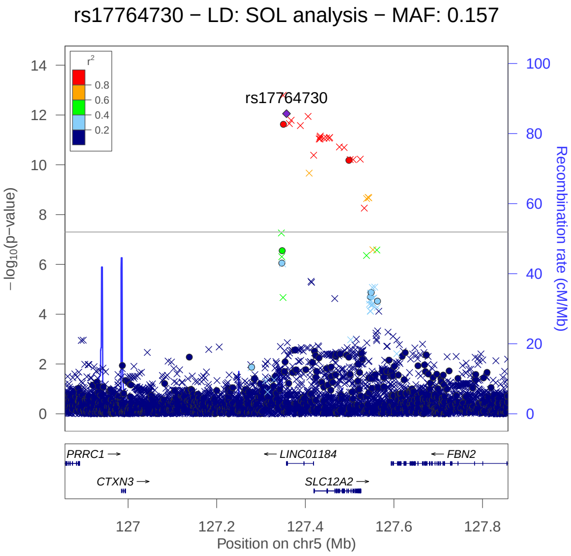

(c) Locus:SLC12A2, lead variant:rs17764730

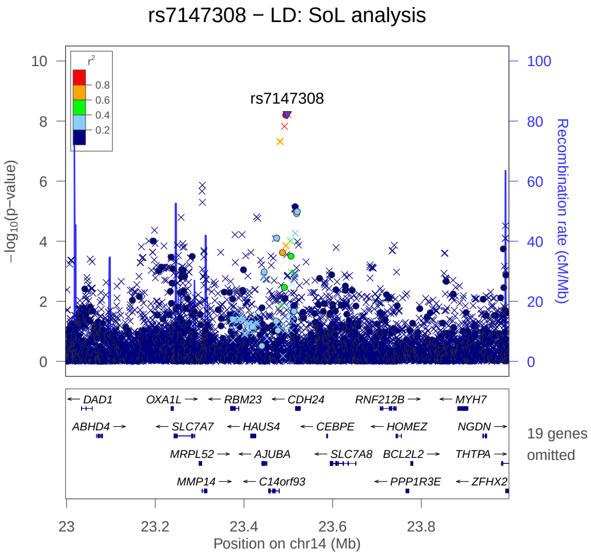

(d) Locus:PSMB5, lead variant:rs7147308

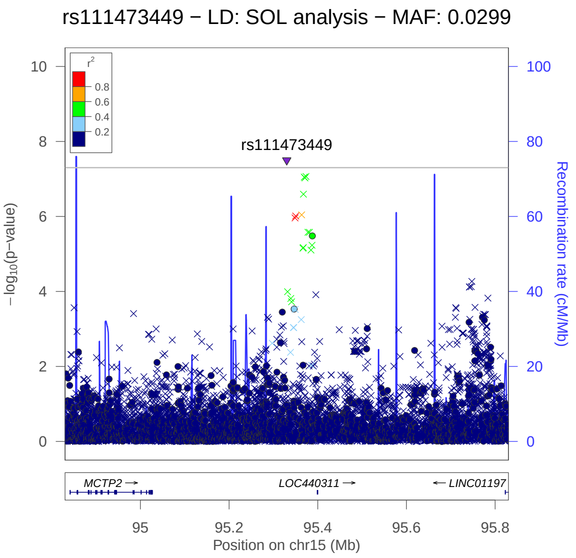

(e) Locus:MCTP2, lead variant:rs111473449

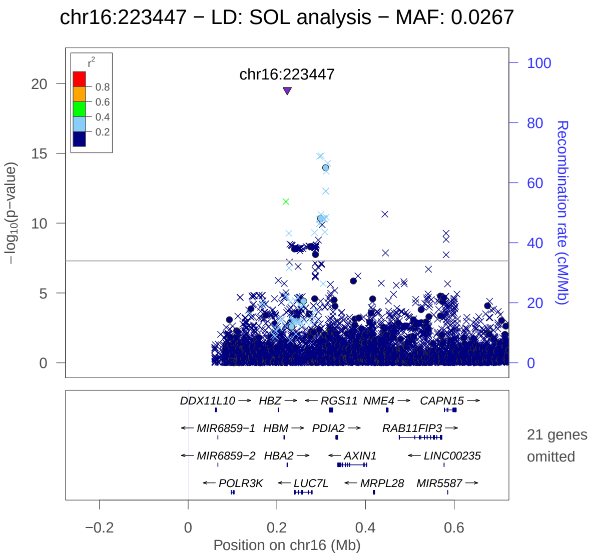

(f) Locus:HBA1/HBA2, lead variant:esv2676630

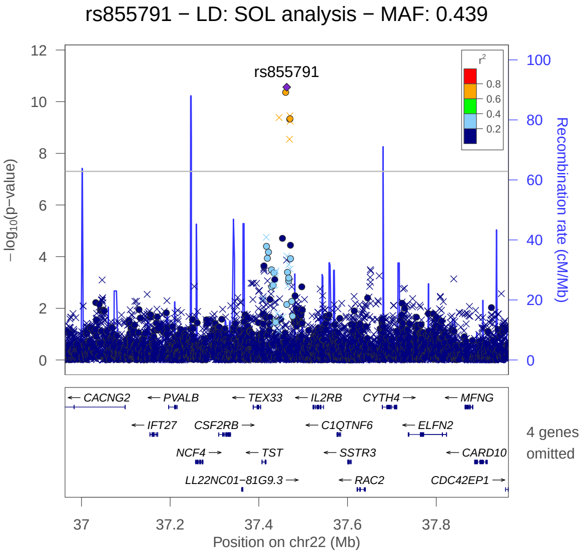

(g) Locus:TMPRSS6, lead variant:rs855791

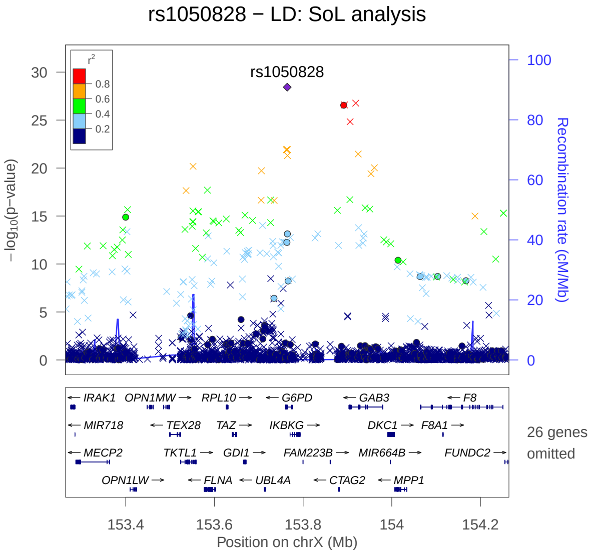

(h) Locus:G6PD, lead variant:rs1050828

Fig S2.5 MCH

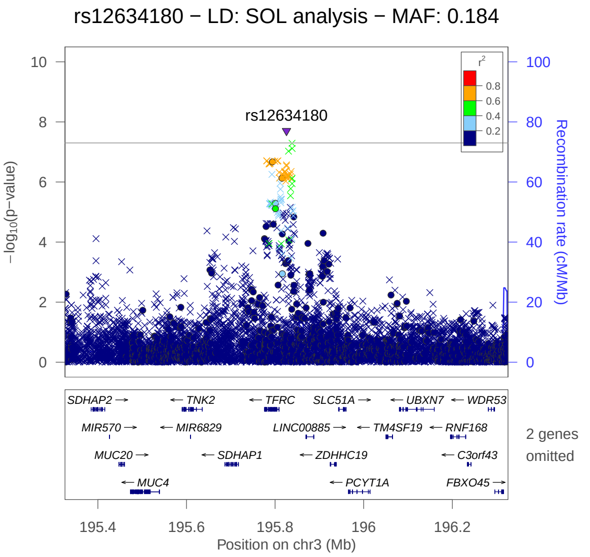

(a) Locus:TFRC, lead variant:rs12634180

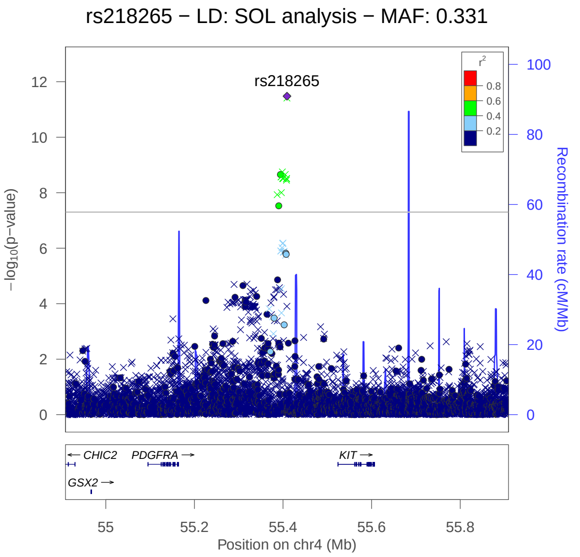

(b) Locus:KIT, lead variant:rs218265

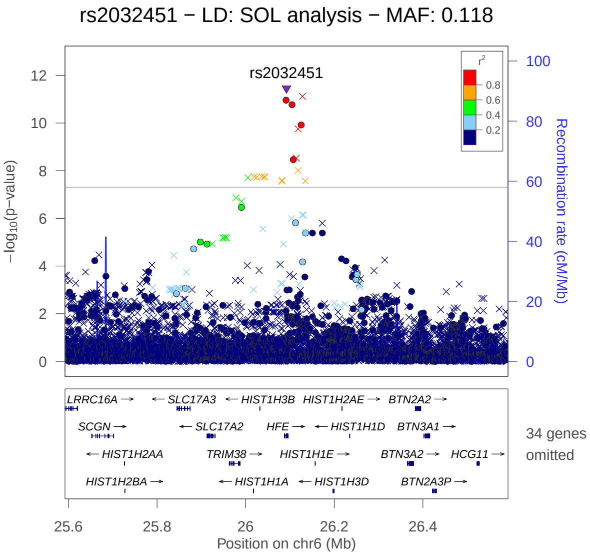

(c) Locus:HFE, lead variant:rs2032451

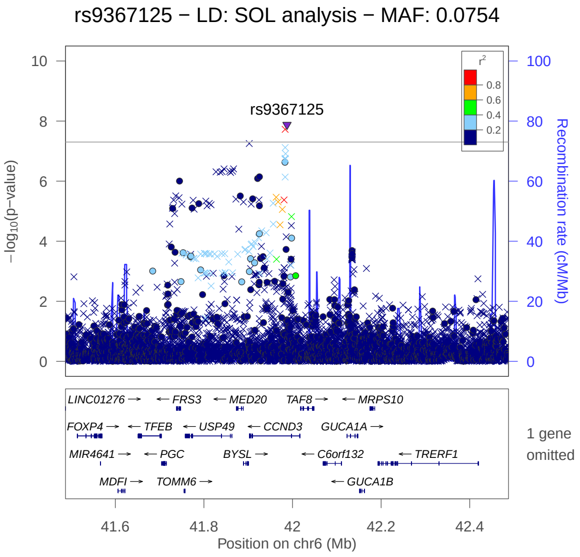

(d) Locus:CCND3, lead variant:rs9367125

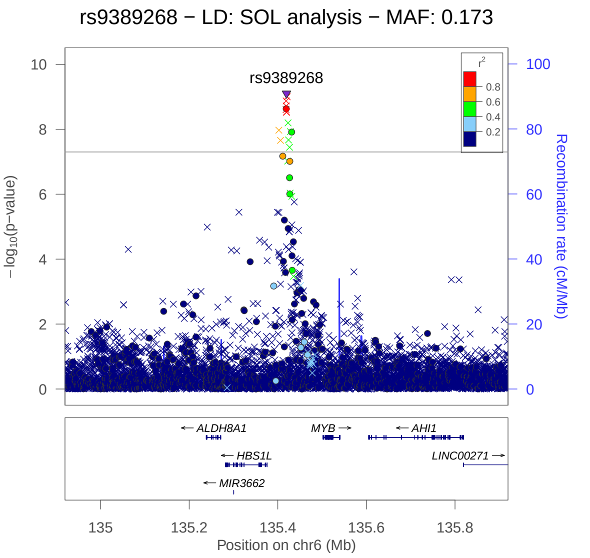

(e) Locus:HBS1L/MYB, lead variant:rs9389268

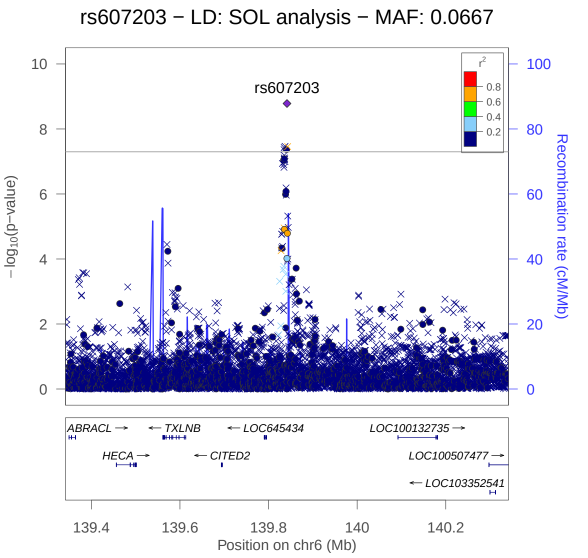

(f) Locus:CITED2, lead variant:rs607203

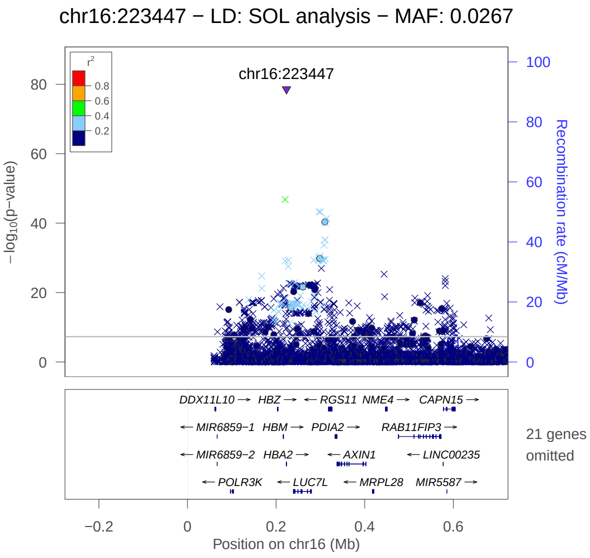

(g) Locus:HBA1/HBA2, lead variant:esv2676630

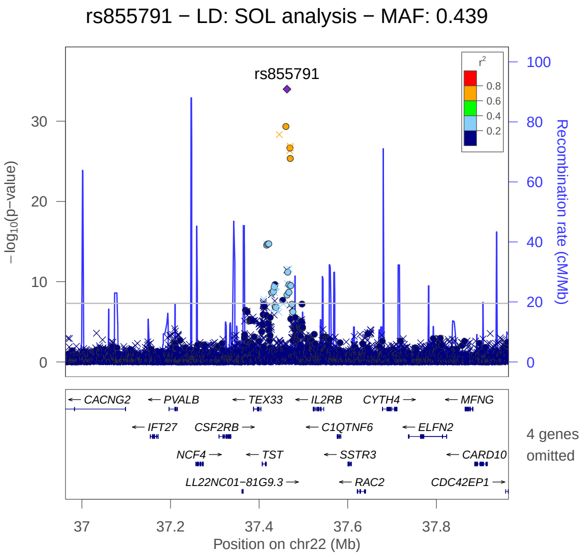

(h) Locus:TMPRSS6, lead variant:rs855791

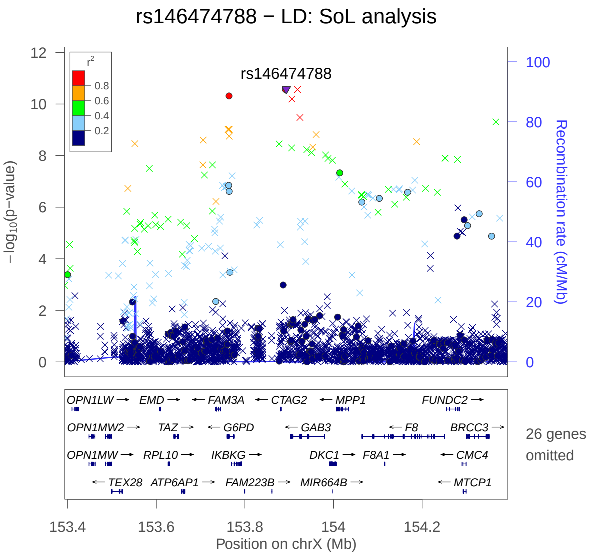

(i) Locus:CTAG2/GAB3, lead variant:rs146474788

Fig S2.6 MCHC

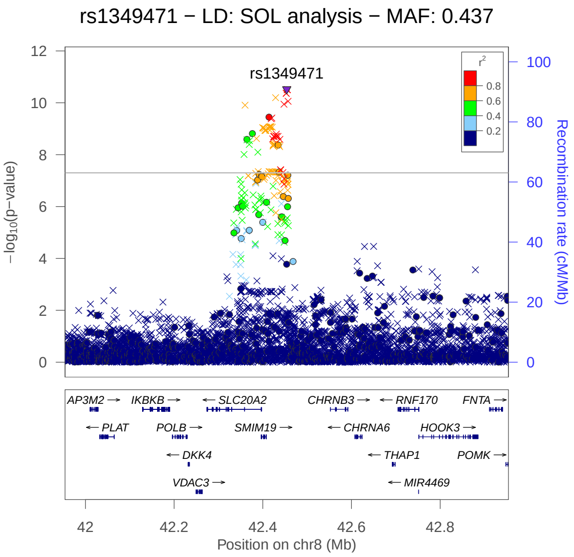

(a) Locus:SMIM19, lead variant:rs1349471

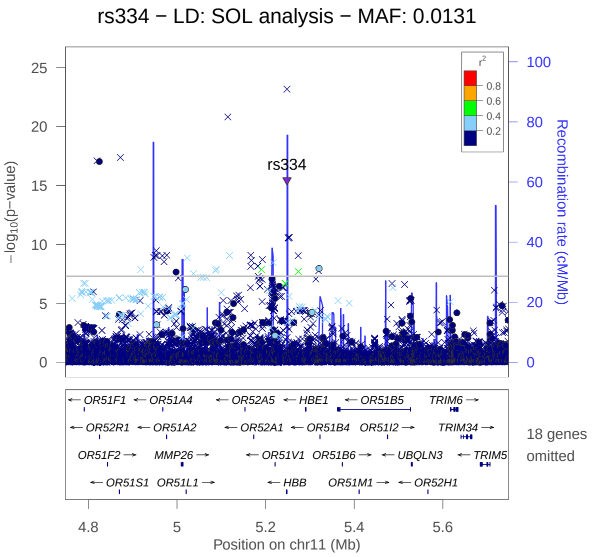

(b) Locus:HBB, lead variant:rs334

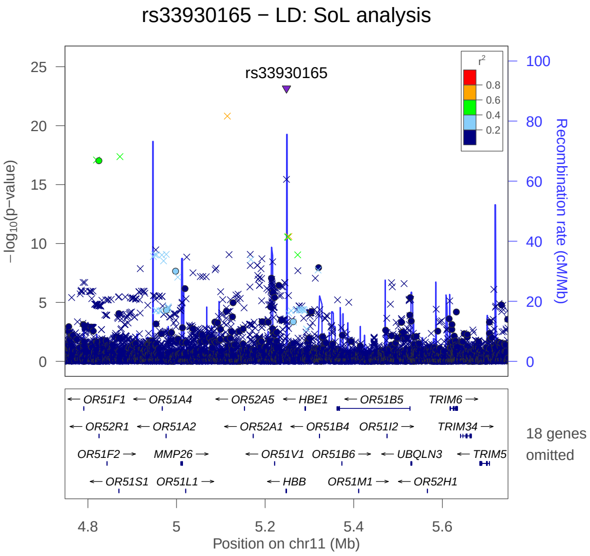

(c) Locus:HBB, lead variant:rs33930165

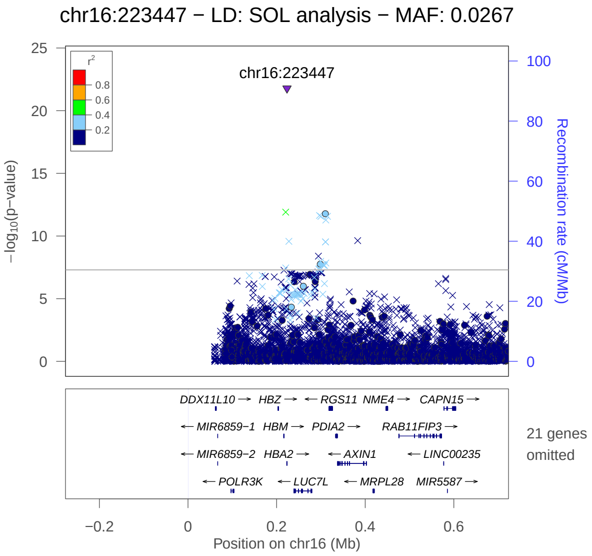

(d) Locus:HBA1/HBA2, lead variant:esv2676630

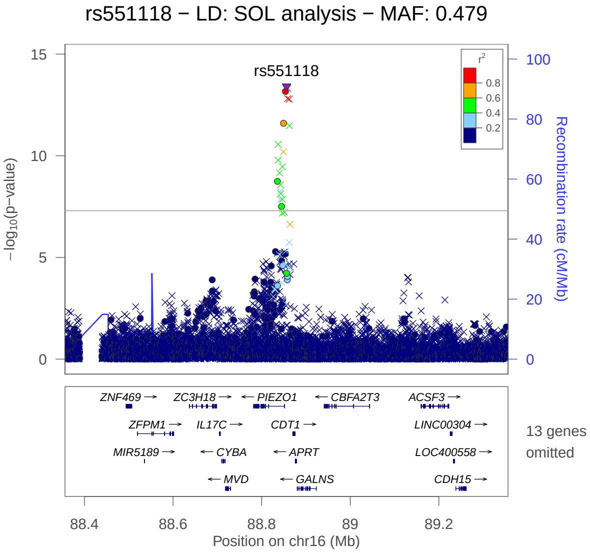

(e) Locus:PIEZO1, lead variant:rs551118

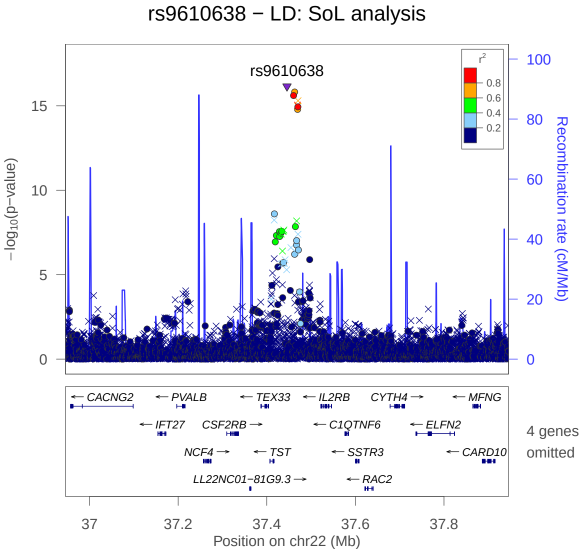

(f) Locus:KCTD17, lead variant:rs9610638

Fig S2.7 MCV

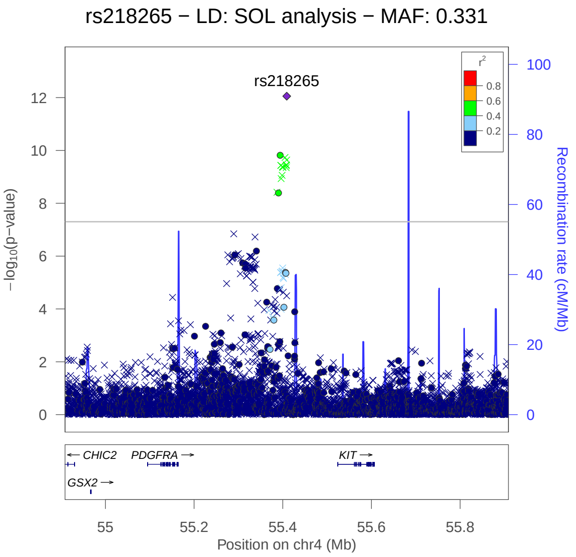

(a) Locus:KIT, lead variant:rs218265

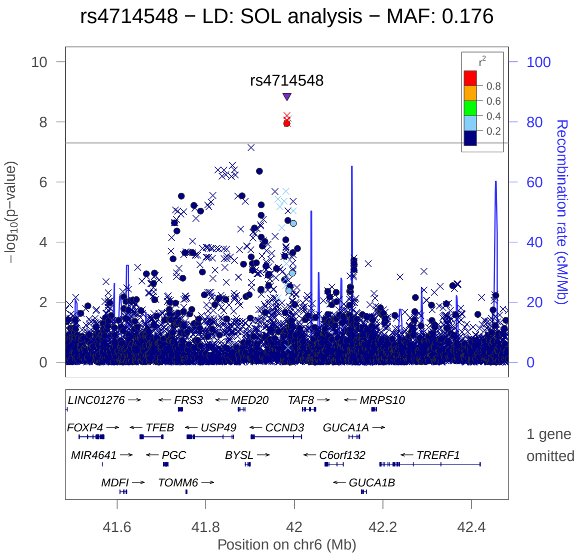

(b) Locus:CCND3, lead variant:rs4714548

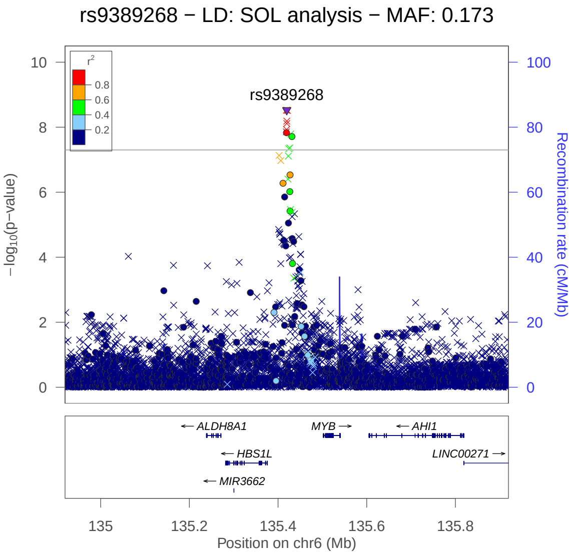

(c) Locus:HBS1L/MYB, lead variant:rs9389268

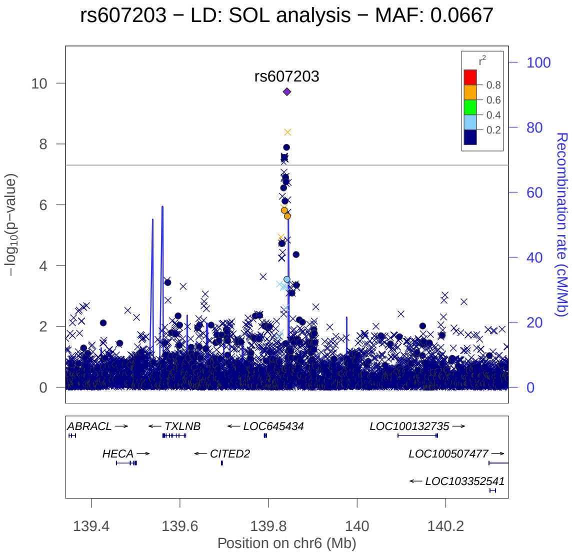

(d) Locus:CITED2, lead variant:rs607203

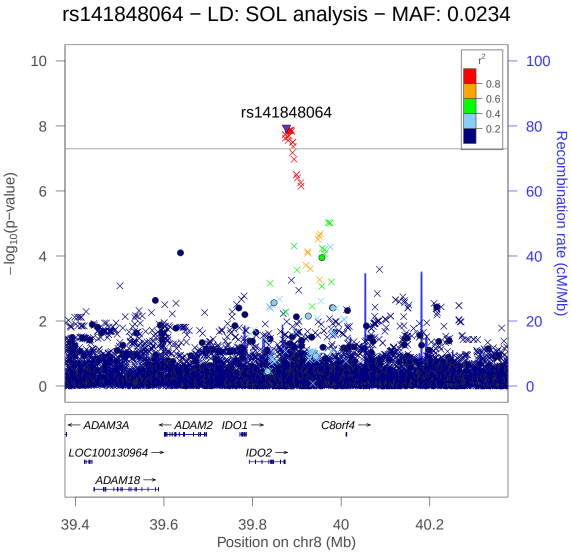

(e) Locus:IDO2, lead variant:rs141848064

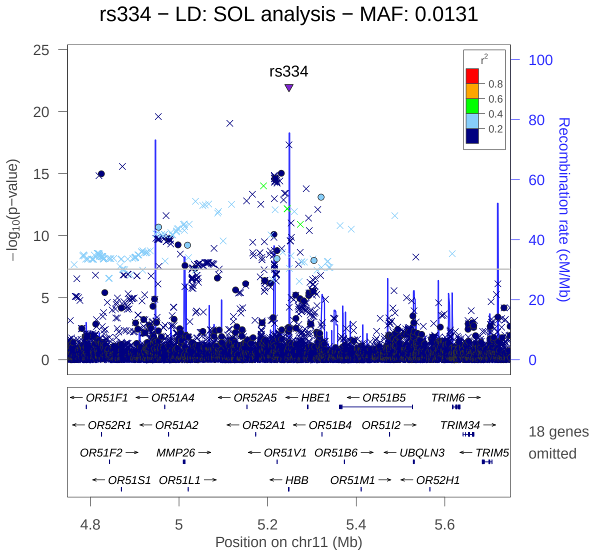

(f) Locus:HBB, lead variant:rs334

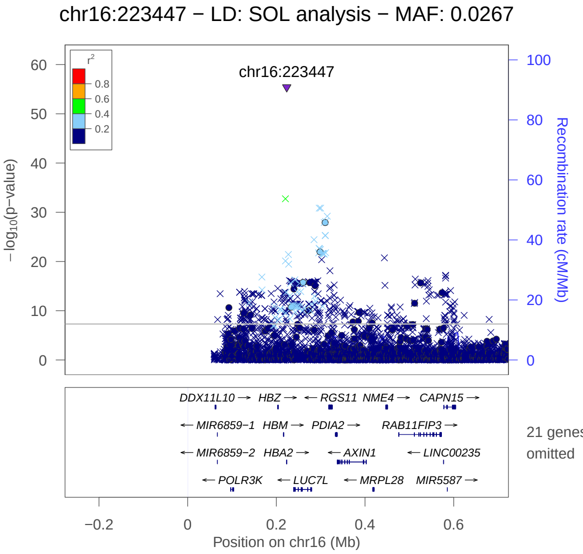

(g) Locus:HBA1/HBA2, lead variant:esv2676630

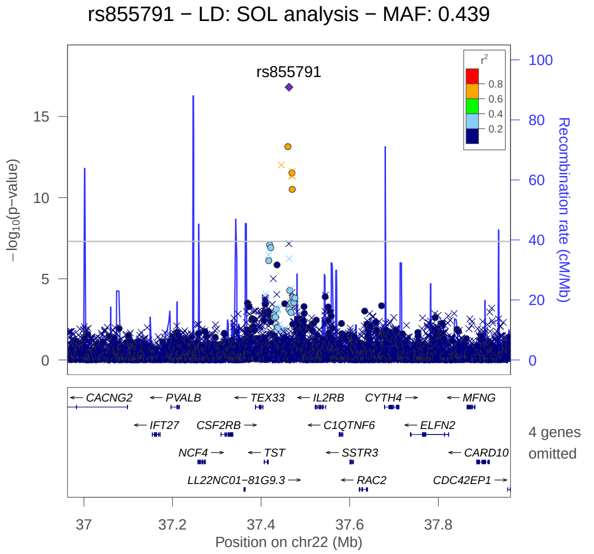

(h) Locus:TMPRSS6, lead variant:rs855791

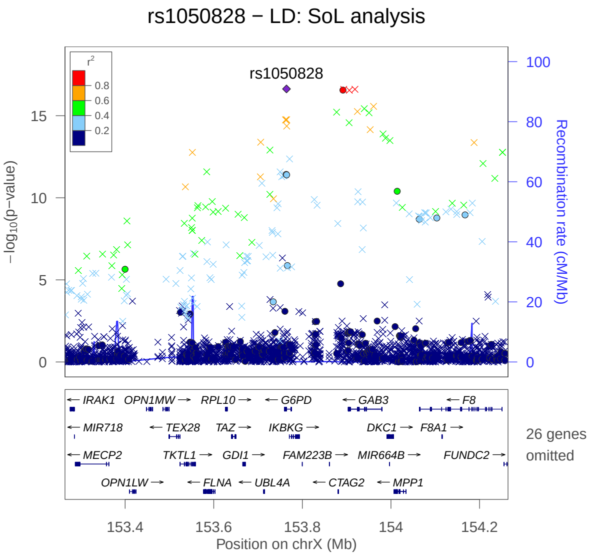

(i) Locus:G6PD, lead variant:rs1050828
