# Supplementary figures and images for: Genome-wide association study of red blood cell traits in Hispanics/Latinos: The Hispanic Community Health Study/Study of Latinos
Source: PLoS Genet. 2017 Apr 28;13(4):e1006760. doi: 10.1371/journal.pgen.1006760 (PMC5428979; doi:10.1371/journal.pgen.1006760)

**Figure S3.** Impact of Deletion of LINC01184 Exon-3 On Expression of LINC01184 and SLC12A2.


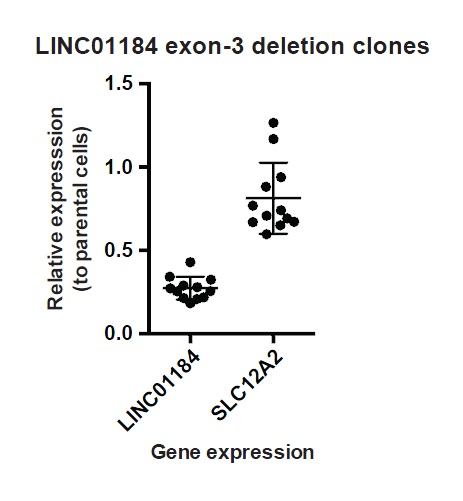

Supplement: S3 Fig — HUDEP-2 human erythroid precursor cells were transduced with lentivirus expressing Cas9 and a pair of guide RNAs targeting cleavages flanking exon-3 of LINC01184. After limiting dilution, clones were screened by PCR for deletion of LINC01184 exon-3. Twelve clones with biallelic deletion of LINC01184 exon-3 were identified and utilized for quantitative reverse transcription PCR to measure expression of LINC01184 and SLC12A2. Primers for LINC01184 measurement annealed to sequences at exons 1 and 2, i.e., non-deleted sequences. Data is shown for each of 12 biallelic deletion clones performed in technical triplicate. Gene expression is normalized to the level of parental cells. Lines indicate means and standard deviations. (DOCX) [file pgen.1006760.s003.docx]

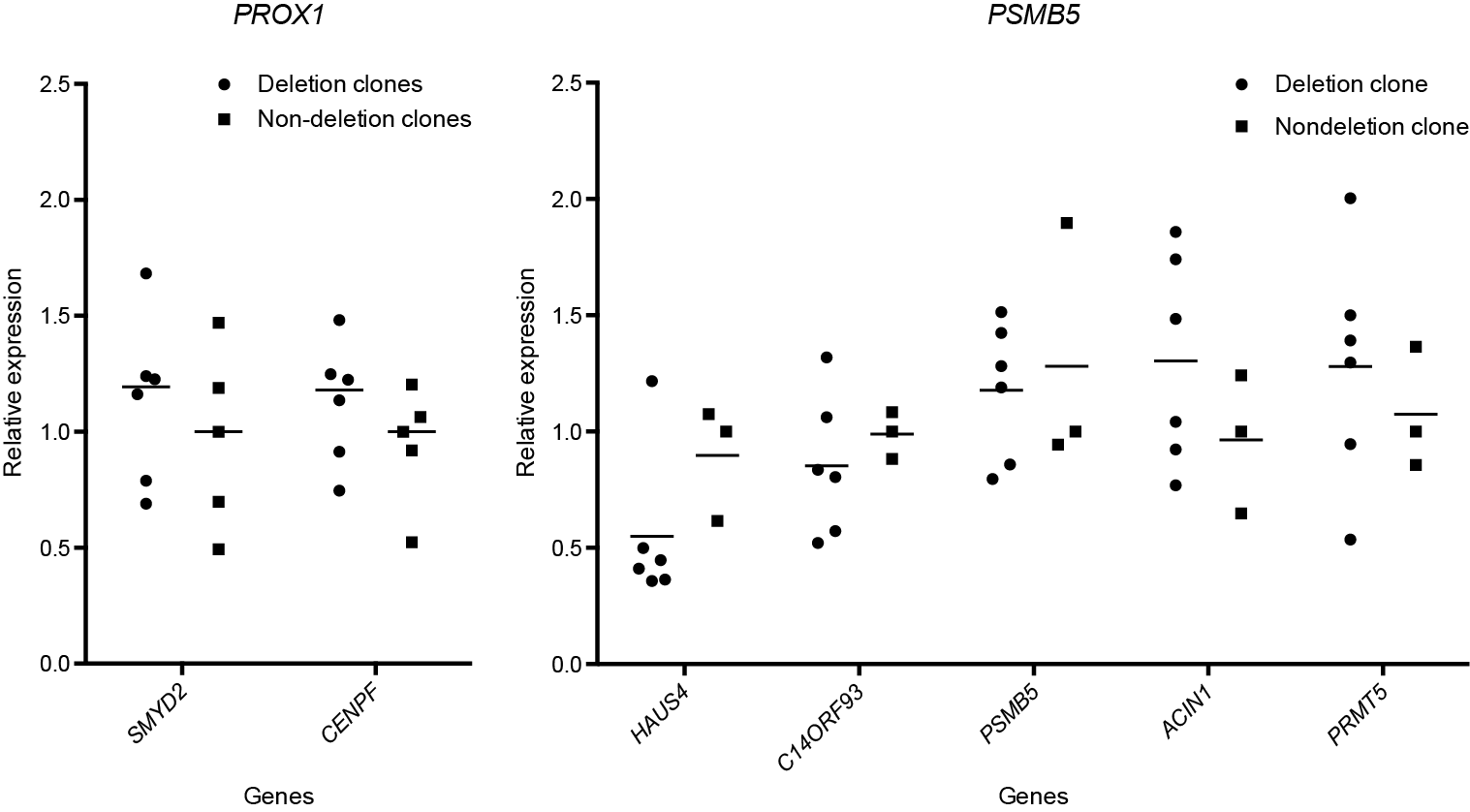

Supplement: S4 Fig — Deletions of DNase I hypersensitive sites (DHSs) at PSMB5 and PROX1 loci were not associated with significant gene expression changes in cis. HUDEP-2 human erythroid precursor cells were transduced with lentivirus expressing Cas9 and a pair of guide RNAs targeting cleavages flanking DHSs at PSMB5 and PROX1. After limiting dilution, clones were screened by PCR for deletion of DHSs. Biallelic deletion clones were identified and utilized for quantitative reverse transcription PCR to measure expression of neighboring genes. As a control, nondeletion clones were isolated in parallel. Data is shown for RT-qPCR for indicated gene in a single clone, normalized to GAPDH, and then to median of the nondeletion clones. Each measurement was performed in technical triplicate. Lines indicate medians of each set of clones. No significant differences were identified between deletion and nondeletion clones (p > 0.05 for all comparisons). (TIF) [file pgen.1006760.s004.tif]
