# Supplementary material for: Genome-wide association study of red blood cell traits in Hispanics/Latinos: The Hispanic Community Health Study/Study of Latinos
Source: PLoS Genet. 2017 Apr 28;13(4):e1006760. doi: 10.1371/journal.pgen.1006760 (PMC5428979; doi:10.1371/journal.pgen.1006760)
Supplement: S5 Fig — X-axis of Manhattan plots = ordered autosomal chromosomes; Y-axis of Manhattan plots = -log10(p-value). The X chromosome was not evaluated because established methods for admixture mapping of this chromosome are not available. (DOCX) [file pgen.1006760.s005.docx]

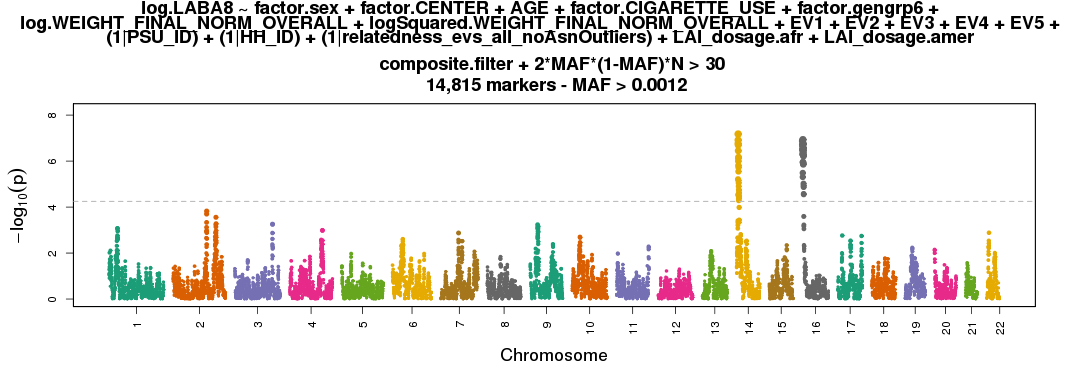

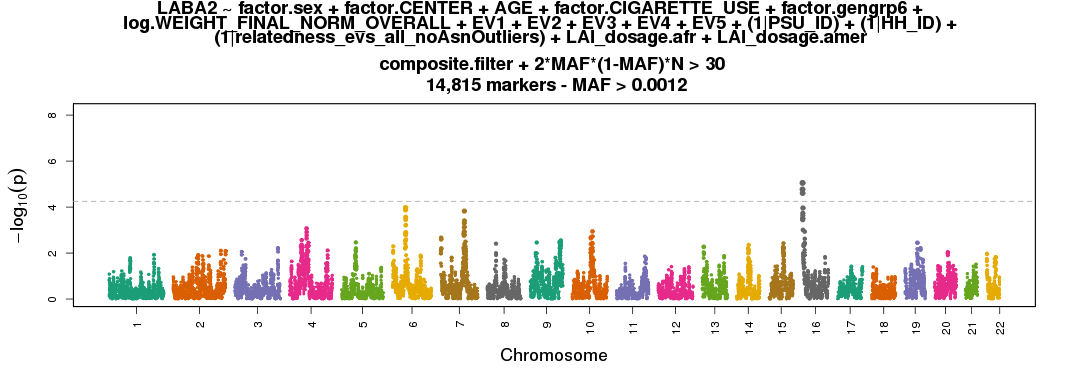

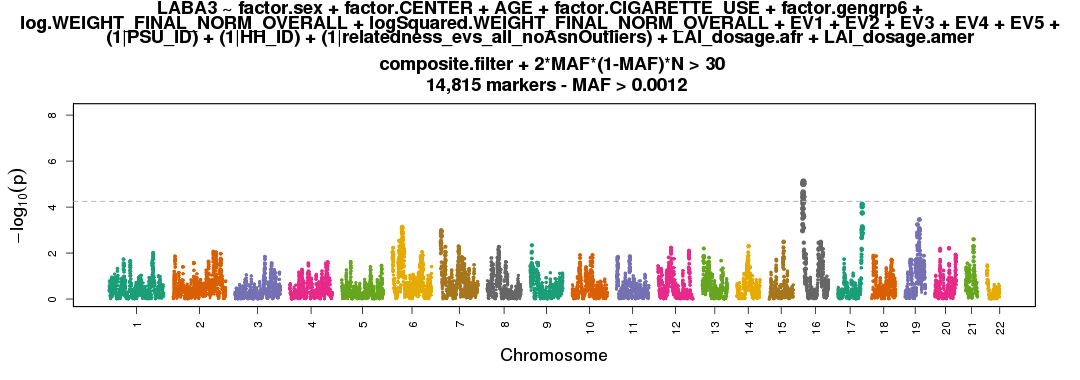


**Figure S5.** Manhattan plots from admixture mapping analysis of RBC Traits in HCSC/SOL participants.


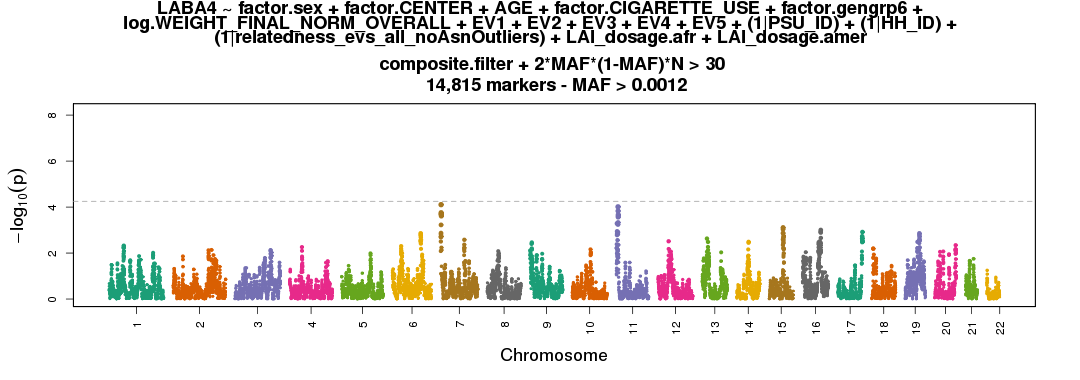


**Hematocrit**

**Hemoglobin**

**Red Blood Cell Count**

**Red Cell Distribution Width**


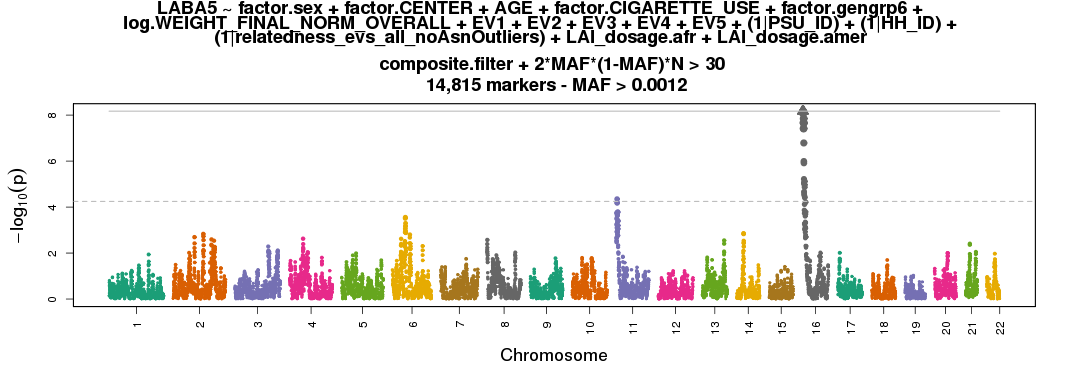

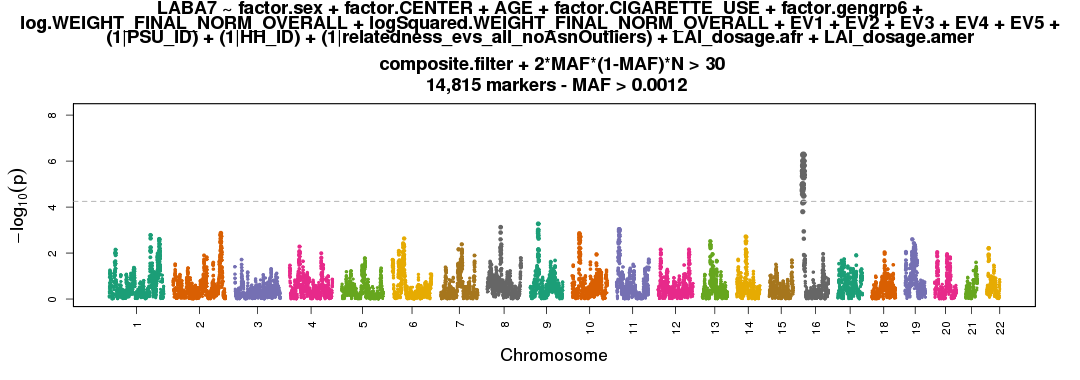

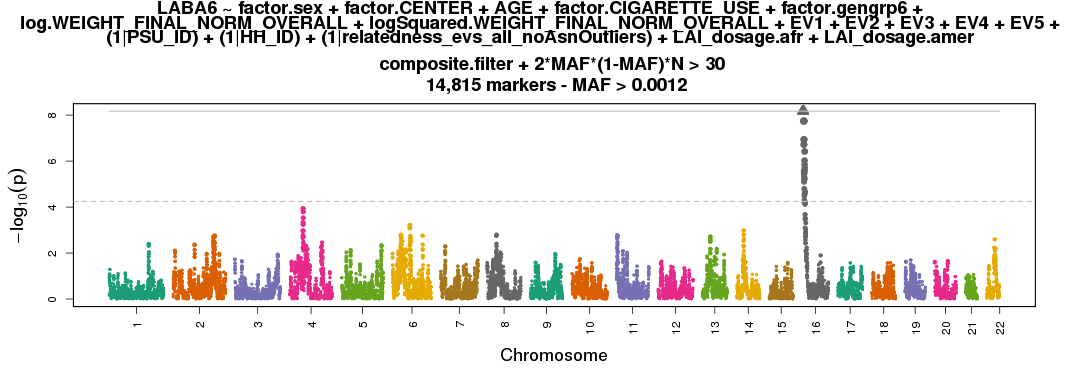


**Mean Corpuscular Hemoglobin Concentration**

**Mean Corpuscular Hemoglobin**

**Mean Corpuscular Volume**
