# Supplementary material for: Genome-wide association study of red blood cell traits in Hispanics/Latinos: The Hispanic Community Health Study/Study of Latinos
Source: PLoS Genet. 2017 Apr 28;13(4):e1006760. doi: 10.1371/journal.pgen.1006760 (PMC5428979; doi:10.1371/journal.pgen.1006760)
Supplement: S1 Table — Genomic inflation factor refers to the ratio between the median test statistics value and the expected median for variants with MAF ≥ 0.01. (DOCX) [file pgen.1006760.s006.docx]

| **S1 Table.** Red Blood Cell Trait Descriptions. | | | | | | |
| --- | --- | --- | --- | --- | --- | --- |
| **Trait** | **Abbreviation** | **Definition** | **Unit** | **Transformation** | **Calculation** | **Genomic inflation factor** |
| Hematocrit | HCT | RBCs as proportion of total blood volume | % | N/A | N/A | 1.018 |
| Hemoglobin | HGB | Concentration of hemoglobin per dL of whole blood | g/dL | N/A | N/A | 1.026 |
| Red blood cell count | RBC count | # of RBCs per mL of whole blood | cells x 10^9^ | N/A | N/A | 1.048 |
| Red cell distribution width (coefficient of variation) | RDW-CV | Width of intra-individual volume curve of red blood cell size | % | Natural log | $\frac{RDW\left( SD \right)\times100}{MCV}$ | 1.054 |
| Mean Corpuscular Hemoglobin | MCH | Average mass of hemoglobin per RBC | pg | N/A | $\frac{HGB\times10}{RBC}$ | 1.045 |
| Mean Corpuscular Hemoglobin Concentration | MCHC | Average concentration of hemoglobin per standard unit of packed RBCs | g/dL | N/A | $\frac{HGB\times100}{HCT}$ | 1.015 |
| Mean Corpuscular Volume | MCV | Average volume of one RBC | fL | N/A | $\frac{HCT\times10}{RBC}$ | 1.044 |
