# Supplementary material for: Genome-wide association study of red blood cell traits in Hispanics/Latinos: The Hispanic Community Health Study/Study of Latinos
Source: PLoS Genet. 2017 Apr 28;13(4):e1006760. doi: 10.1371/journal.pgen.1006760 (PMC5428979; doi:10.1371/journal.pgen.1006760)
Supplement: S2 Table — *Units for each trait are as follows: Hematocrit, %; Hemoglobin, g/dL; RBC count, cells x109; RDW, %; MCH, pg; MCHC, g/dL; MCV, fL. Population means for hematocrit and hemoglobin are presented as sex-stratified due to significant differences between adult males and females. ** Hematocrit and hemoglobin were available at the baseline exam for WHI SHARe in 3,539 participants. The remaining measures were available in a sub-sample of 1,205 WHI SHARe participants. (DOCX) [file pgen.1006760.s007.docx]

| **S2 Table.** Characteristics of Discovery and Replication Cohorts. | | | | | | |  |  |  |  |  |  |
| --- | --- | --- | --- | --- | --- | --- | --- | --- | --- | --- | --- | --- |
| **Study** | **Phase** | **N** | **Study Design** | **Mean HCT* (SD)** | **Mean HGB* (SD)** | **Mean RBC* Count (SD)** | **Mean RDW* (SD)** | **Mean MCH* (SD)** | **Mean MCHC* (SD)** | **Mean MCV* (SD)** | **% Female** | **Mean Age (SD)** |
| HCHS/SOL | Discovery | 12,502 | Population- and family-based | F: 40.2 (3.4) | F: 13.0 (1.2) | 4.73 (0.44) | 13.7 (1.3) | 29.2 (2.2) | 32.7 (1.5) | 89.2 (5.9) | 59 | 46.1 (13.8) |
|  |  |  |  | M: 44.8 (3.4) | M: 14.9 (1.2) |  |  |  |  |  |  |  |
| BioMe | Replication | 2,785 | Population-based, unrelated | F: 37.9 (3.7) | F: 12.7 (1.3) | 4.40 (0.53) | 14.3 (1.7) | 30.1 (2.5) | 33.6 (0.8) | 89.5 (6.4) | 62 | 59.5 (15.3) |
|  |  |  |  | M: 41.4 (4.5) | M: 14.0 (1.6) |  |  |  |  |  |  |  |
| WHI SHARe | Replication | 3,539** | Population-based, unrelated | F: 39.9 (2.8) | F: 13.4 (0.9) | 4.42 (0.40) | 13.9 (1.1) | 30.0 (1.9) | 33.2 (1.1) | 90.3 (5.1) | 100 | 60 (7.0) |
| MESA | Replication | 782 | Population-based, unrelated | F: 38.8 (3.5) | F: 12.8 (1.2) | 4.49 (0.49) | 14.2 (1.2) | 30.0 (2.4) | 33.2 (1.2) | 90.6 (5.6) | 53 | 69 (9.0) |
|  |  |  |  | M: 42.4 (3.9) | M: 14.2 (1.4) |  |  |  |  |  |  |  |
