# Supplementary material for: Genome-wide association study of red blood cell traits in Hispanics/Latinos: The Hispanic Community Health Study/Study of Latinos
Source: PLoS Genet. 2017 Apr 28;13(4):e1006760. doi: 10.1371/journal.pgen.1006760 (PMC5428979; doi:10.1371/journal.pgen.1006760)
Supplement: S5 Table — C/A = coded and alternate alleles. CAF = coded allele frequency. (DOCX) [file pgen.1006760.s010.docx]

| **S5 Table.** Summary of findings from the functional annotation of novel red blood cell-associated variants and their LD partners (r^2^ ≥0.8) identified in HCHS/SOL. | | | | | | | | | | | | | | | | | | |
| --- | --- | --- | --- | --- | --- | --- | --- | --- | --- | --- | --- | --- | --- | --- | --- | --- | --- | --- |
| **Trait** | **Index SNP rsID** | **fSNP^*^ rsID** | **r^2^** | **Functional SNP** | | | | | | | | **fSNP 1000 genomes phase III CAF** | | | | | | **Description of Findings** |
|  |  |  |  | **p-value** | | **Beta (SE)** | | **Nearest Genes to** | **C / A allele** | | **CAF** | **AFR** | **EUR** | **AMR** | **SAS** | **EAS** | **All** |  |
|  | rs144261491 | rs200572016 | 0.8 |  | 0.393 (0.081) | | *MEF2C-AS1* | | | TGA/T | 0.97 | 1 | 1 | 0.96 | 1 | 1 | 0.99 | indel rs200572016 lies in a putative megakaryocyte-specific enhancer in *MEF2C-AS1* intron; this enhancer element is DNAse accessible in K562 cells and overlaps ChIP-Seq peaks of GATA2, TAL1 and P300 in these cells |
| HCT | rs3754140 | rs7541039 | 1.0 | 4.45E-07 | -0.225  (0.045) | | *PROX1* | | | C/T | 0.62 | 0.73 | 0.74 | 0.55 | 0.83 | 0.69 | 0.72 | There are three functional SNP candidates in this locus namely rs7541039, rs7517701, and rs4282786; all of them are located in the same enhancer element which is positioned in the intronic region of *PROX1*. The enhancer element is DNAse hypersensitive in CD34 derived proerythroblasts and k562 cells and enriched with H3k4me1 mark in k562 cells. It overlaps with GATA1, TAL1 ChIP-Seq peaks in bone marrow derived proerythrobalsts and K562 cells, PolII in k562 cells. |
| HCT | rs3754140 | rs7517701 | 1.0 | 8.45E-08 | -0.238  (0.045) | | *PROX1* | | | G/C | 0.61 | 0.73 | 0.73 | 0.55 | 0.83 | 0.69 | 0.72 |  |
| HCT | rs3754140 | rs4282786 | 1.0 | 8.46E-08 | -0.238  (0.045) | | *PROX1* | | | G/A | 0.61 | 0.73 | 0.73 | 0.55 | 0.83 | 0.69 | 0.72 |  |
| RDW | rs941718 | rs11846575 | 1.0 | 1.50E-08 | -0.007 (0.001) | | *PSMB5* | | | C/G | 0.70 | 0.17 | 0.70 | 0.79 | 0.60 | 0.94 | 0.60 | There are not any strong functional candidates in this locus; however, tag SNP rs11846575 is a potential functional candidate. rs11846575 lies in the intergenic region between *PSMB5* and non-coding RNA *SNORA73*. rs11846575 overlaps with enhancer element which is enriched for H3k4me1 mark in K562 cells. The enhancer element is bound by several erythroid TFs namely, GATA1, TAL1, and SMAD1 in erythroblasts. rs11846575 is reported as an eQTL for *PRMT5-AS1*, *PRMT5*, and *HAUS4*. It is also predicted to disrupt ETS, PAX5, and TATA DNA-binding motifs. |
| RDW | rs17764730 | rs3812049 | 0.9 | 4.13E-11 | -0.009  (0.001) | | *SLC12A2/ CTC-228N24.3* | | | C/G | 0.83 | 0.09 | 0.21 | 0.16 | 0.37 | 0.39 | 0.24 | rs3812049 lies in a promoter element in between genes *SLC12A*2 and non-coding RNA *CTC-228N24.3*. The promoter element harboring rs3812049 is DNAse hypersensitive in CD34-derived proerythroblasts and K562 cells. It is also enriched for the histone marks H3k27ac and H3k4me3, H3k4me1 in erythroblasts^1^. Furthermore, the promoter element overlaps with GATA1 and TAL1 peaks in bone marrow derived proerythroblasts, POLII and CHD2, TAF1, CTCF and other transcription factors in K562 cells. rs3812049 is also reported as an eQTL for CTC-228N24.3 in various tissues including whole blood. |
