# Supplementary material for: Genome-wide association study of red blood cell traits in Hispanics/Latinos: The Hispanic Community Health Study/Study of Latinos
Source: PLoS Genet. 2017 Apr 28;13(4):e1006760. doi: 10.1371/journal.pgen.1006760 (PMC5428979; doi:10.1371/journal.pgen.1006760)
Supplement: S7 Table — F = forward, R = reverse. Chromosomal positions refer to hg build19/GRCh37. (DOCX) [file pgen.1006760.s012.docx]

| **S7 Table.** Oligonucleotide Sequences used in CRISPR-Cas9 Genome Editing, PCR screening and RT-qPCR quantification. | | | |
| --- | --- | --- | --- |
| **Genomic Region** | **Oligonucleotide type** | **Oligonucleotide name** | **Sequence** |
| ***PROX1 DHS deletion.***  ***Genomic coordinates of edited loci (hg19) chr1:214,176,510-214,177,217*** | Guide RNA sequences | sgRNA-PROX1-DHS-5’ | TTAAAAGCTTCAGGAAGTTT |
|  |  | sgRNA-PROX1-DHS-3’ | AGTCACGCTAGACTTGATGA |
|  | *Gap PCR primers* | PROX1-DHS-gap-out-F | CAGTTCCAAGTATTACCAACAGGA |
|  |  | PROX1-DHS-gap-out-R | CCCCTTAGGCAGAAATGTCA |
|  |  | PROX1-DHS-inner-F | TATCACAAGGCAGTCGCAGA |
|  |  | PROX1-DHS-inner-R | GCCAAAGCAGCTTAACTGAA |
|  | RT-qPCR primers | PROX1-RT-e4-F | TCTGAACATGCACTACAATAAAGC |
|  |  | PROX1-RT-e5-R | GATCAACATCTTTGCCTGCG |
|  |  | SMYD2-RT-e11-F | GGGAGAAAGCCCTGAAGAAG |
|  |  | SMYD2-RT-e12-R | TTCTGAACTAAGTGTTTAAATGAAAACTG |
|  |  | CENPF-RT-e2-F | CAAAGGCAGTTTCAGCTTGAC |
|  |  | CENPF-RT-e3-R | GCTTAGTTTTCTCCAGACTTTCAC |
| ***SLC12A2* - *LINC01184* promoter *indels. Genomic coordinates of edited loci (hg19) chr5:127418849-127418850 (HUDEP-2 genotype is rs3812049-G/G)*** | Guide RNA sequence | sgRNA-SLC12A2-prom | TGCGAGTTCACCAAAGGGGG |
|  | *Gap PCR primers* | SLC12A2-prom-F-in | AGCATCGCCCGAAGGAAT |
|  |  | SLC12A2-prom-R-in | CTCACGCCGGTAGCTGAT |
|  |  | SLC12A2-prom-F-out | AGAGCCCAGAGCAAACCAG |
|  |  | SLC12A2-prom-R-out | GCGGCTACCTCCTCTTCC |
|  | RT-qPCR primers | SLC12A2-RT-e20-F | AGAGAAATCTCCTGGCACCAA |
|  |  | SLC12A2-RT-e20-R | CCTCTTCCTCAACTTTGTGTGT |
| ***LINC01184 exon3 deletion.***  ***Genomic coordinates of edited loci (hg19) chr5:127,356,732-127,360,544*** | Guide RNA sequence | sgRNA-LINC01184-e3-5’ | TGTTAAACAGAGGAGCCACC |
|  |  | sgRNA-LINC01184-e3-3’ | AGCGCTAGGTGATGAAGGCT |
|  | *Gap PCR primers* | LINC01184-gap-out-F | TTCTCCTCTCCTGCCTCAAA |
|  |  | LINC01184-gap-out-R | TCCAGGAACTGCATCTTCAA |
|  |  | LINC01184-gap-inner-F | TTGTGCCTTACCCTTTTTGG |
|  |  | LINC01184-gap-inner-R | AGCTGCTACATCGGACAGGT |
|  | RT-qPCR primers | LINC01184-RT-e1-F | AGTGAGGCTGCCATAAATCTC |
|  |  | LINC01184-RT-e1-R | ATTCCTTCGGGCGATGC |
| ***PSMB5 DHS deletion.***  ***Genomic coordinates of edited loci (hg19) chr14:23,489,921-23,490,427*** | Guide RNA sequence | sgRNA-PSMB5-DHS-5’ | ATCAATCCAAGTGCGGTCCT |
|  |  | sgRNA-PSMB5-DHS-3’ | GGGCCTAGGGTGCCTAAGTC |
|  | *Gap PCR primers* | PSMB5-DHS-gap-out-F | TGCCCAGATGGTCTTGAACT |
|  |  | PSMB5-DHS-gap-out-R | CCCTGGCAACACACATCATA |
|  |  | PSMB5-DHS-inner-F | GACTCAAGGGAGACATGGTGA |
|  |  | PSMB5-DHS-inner-R | CCGCAGCTCTTTTCTCATTC |
|  | RT-qPCR primers | PSMB5-RT-e2-F | ATGATCTGTGGCTGGGATAAG |
|  |  | PSMB5-RT-e3-R | CATACACAGAGCCAGAACCTAC |
|  |  | PRMT5-RT-e2-F | CCAACCACATCCACACTGG |
|  |  | PRMT5-RT-e3-R | ACCACATCCACGTTTTCTCC |
|  |  | HAUS4-RT-e1-F | GCATCCGGGGATTTCTGCTC |
|  |  | HAUS4-RT-e2-R | GCTGAAGTATGGGTTCTGTAACA |
|  |  | C14ORF93-RT-e4-F | ACCAGTCCCCACAATTACAC |
|  |  | C14ORF93-RT-e5-R | GTACTCACGCCTCTTAGTAAGG |
|  |  | ACIN1-RT-e6-F | AGAAGTGTGAAGCTGAAGAGG |
|  |  | ACIN1-RT-e7-R | GTCTGTTTTCACTTGTGTCCATG |
| **GAPDH** | RT-qPCR primers | GAPDH_RT_125_F | ACCCAGAAGACTGTGGATGG |
|  |  | GAPDH_RT_125_R | TTCAGCTCAGGGATGACCTT |
| **Non target** | Guide RNA sequence | sgRNA-nontargeting | ACGGAGGCTAAGCGTCGCAA |
