# Supplementary material for: Genome-wide association study of red blood cell traits in Hispanics/Latinos: The Hispanic Community Health Study/Study of Latinos
Source: PLoS Genet. 2017 Apr 28;13(4):e1006760. doi: 10.1371/journal.pgen.1006760 (PMC5428979; doi:10.1371/journal.pgen.1006760)
Supplement: S8 Table — * value of 0 = 0 copies of 3.8kb deletion, 1 = 1 copy of deletion, 2 = 2 copies of deletion. (DOCX) [file pgen.1006760.s013.docx]

| **S8 Table.** Comparison of 1000 Genomes phase I and re-typed (based on probe intensity) deletion genotype calls for the alpha globin 3.8kb deletion. | | | |
| --- | --- | --- | --- |
| **Imputed 3.8kb deletion call*** | **re-typed 3.8kb deletion call*** | **Number of samples** |  |
| 0 | 0 | 11,275 |  |
| 1 | 0 | 189 |  |
| 2 | 0 | 3 |  |
| 0 | 1 | 484 |  |
| 1 | 1 | 389 |  |
| 2 | 1 | 3 |  |
| 0 | 2 | 13 |  |
| 1 | 2 | 21 |  |
| 2 | 2 | 13 |  |
|  | | | |
