# Supplementary material for: Genome-wide association study of red blood cell traits in Hispanics/Latinos: The Hispanic Community Health Study/Study of Latinos
Source: PLoS Genet. 2017 Apr 28;13(4):e1006760. doi: 10.1371/journal.pgen.1006760 (PMC5428979; doi:10.1371/journal.pgen.1006760)
Supplement: S10 Table — Chromosomal positions are aligned to build hg19/GRCh37. Alt = alternative; CAF = coded allele frequency; MCH = mean corpuscular hemoglobin; MCV = mean corpuscular volume; RBC = red blood cell count; RDW = red cell distribution width; SE = standard error. (DOCX) [file pgen.1006760.s015.docx]

| **S10 Table.** Sex-stratified results for genome-wide significant X-chromosome associations. | | | | | | | | | |  |
| --- | --- | --- | --- | --- | --- | --- | --- | --- | --- | --- |
|  | | | | | **Women** | | | **Men** | |  |
| **Trait** | **Annotated Gene(s) (location)** | **rsID** | **Chr: position** | **Coded/Alt allele** | **p-value** | **Beta (SE)** | **CAF** | **p-value** | **Beta (SE)** | **CAF** |
| RBC | *G6PD* (missense) | rs1050828 | chrX: 153764217 | C/T | 1.10E-09 | 0.13 (0.02) | 0.98 | 1.10E-11 | 0.15 (0.02) | 0.98 |
| MCV | *G6PD* (missense) | rs1050828 | chrX: 153764217 | C/T | 8.20E-08 | -1.93 (0.36) | 0.98 | 1.20E-12 | -2.03 (0.29) | 0.98 |
| RDW | *G6PD* (missense) | rs1050828 | chrX: 153764217 | C/T | 3.00E-12 | 0.04 (0.01) | 0.98 | 3.90E-19 | 0.03 (0.004) | 0.98 |
| MCH | *CTAG2 / GAB3* (intergenic) | rs146474788 | chrX: 153893403 | G/A | 3.50E-05 | -0.55 (0.13) | 0.98 | 1.20E-09 | -0.59 (0.10) | 0.98 |
