# Supplementary material for: Genome-wide association study of red blood cell traits in Hispanics/Latinos: The Hispanic Community Health Study/Study of Latinos
Source: PLoS Genet. 2017 Apr 28;13(4):e1006760. doi: 10.1371/journal.pgen.1006760 (PMC5428979; doi:10.1371/journal.pgen.1006760)
Supplement: S11 Table — Chromosomal positions are aligned to build hg19/GRCh37. Alt = alternative; CAF = coded allele frequency; MCH = mean corpuscular hemoglobin; MCV = mean corpuscular volume; RBC = red blood cell count; RDW = red cell distribution width; SE = standard error. (DOCX) [file pgen.1006760.s016.docx]

| **S11 Table.** Genotype-specific association results for lead X chromosome variant in HCHS/SOL female participants. |
| --- |

|  | | | | | **Heterozygous genotype** | | **Homozygous rare genotype** | |
| --- | --- | --- | --- | --- | --- | --- | --- | --- |
| **Trait** | **Annotated Gene(s) (location)** | **rsID** | **chr: position** | **Minor allele** | **p-value** | **Beta (SE)** | **p-value** | **Beta (SE)** |
| RBC | *G6PD* (missense) | rs1050828 | chrX: 153764217 | T | 9.52E-09 | -0.13 (0.02) | 1.60E-02 | -0.25 (0.10) |
| MCV | *G6PD* (missense) | rs1050828 | chrX: 153764217 | T | 1.62E-06 | 1.85 (0.39) | 5.32E-03 | 4.82 (1.73) |
| RDW | *G6PD* (missense) | rs1050828 | chrX: 153764217 | T | 2.62E-08 | -0.03 (0.01) | 8.40E-08 | -0.15 (0.03) |
| MCH | *CTAG2 / GAB3* (intergenic) | rs146474788 | chrX: 153893403 | A | 3.95E-04 | 0.52 (0.15) | 9.20E-03 | 1.66 (0.64) |
