# Supplementary material for: Genome-wide association study of red blood cell traits in Hispanics/Latinos: The Hispanic Community Health Study/Study of Latinos
Source: PLoS Genet. 2017 Apr 28;13(4):e1006760. doi: 10.1371/journal.pgen.1006760 (PMC5428979; doi:10.1371/journal.pgen.1006760)
Supplement: S12 Table — Chromosomal positions are aligned to build hg19/GRCh37. Alt = alternative; CAF = coded allele frequency; MCH = mean corpuscular hemoglobin; MCV = mean corpuscular volume; RBC = red blood cell count; RDW = red cell distribution width; SE = standard error. (DOCX) [file pgen.1006760.s017.docx]

| **S12 Table.** Interaction results of age and lead X chromosome variant genotype in HCHS/SOL female participants. | | | | | | |
| --- | --- | --- | --- | --- | --- | --- |
|  | | | | | | |
|  | | | | | **Age:genotype interaction** | |
| **Trait** | **Annotated Gene(s) (location)** | **rsID** | **Chr: position** | **Coded/Alt allele** | **p-value** | **Beta (SE)** |
| RBC | *G6PD* (missense) | rs1050828 | chrX: 153764217 | C/T | 7.81E-02 | -0.0026 (0.0015) |
| MCV | *G6PD* (missense) | rs1050828 | chrX: 153764217 | C/T | 9.38E-01 | -0.002 (0.0253) |
| RDW | *G6PD* (missense) | rs1050828 | chrX: 153764217 | C/T | 3.03E-01 | 0.0004 (0.0004) |
| MCH | *CTAG2 / GAB3* (intergenic) | rs146474788 | chrX: 153893403 | G/A | 9.27E-01 | -0.0009 (0.0096) |
