# Supplementary material for: Genome-wide association study of red blood cell traits in Hispanics/Latinos: The Hispanic Community Health Study/Study of Latinos
Source: PLoS Genet. 2017 Apr 28;13(4):e1006760. doi: 10.1371/journal.pgen.1006760 (PMC5428979; doi:10.1371/journal.pgen.1006760)
Supplement: S14 Table — EV = Eigenvector; HCT = hematocrit; HGB = hemoglobin; MCH = mean corpuscular hemoglobin; MCHC = MCH concentration; MCV = mean corpuscular volume; RBC = red blood cell count; RDW = red cell distribution width; hba_cnv_countDel = intensity-based calls for the alpha gene deletion; hba_cnv_countDupl = intensity-based calls for the alpha gene duplication. † imputed calls for esv2676630 were used for conditional analyses (see Methods). †† probe intensity-based re-typed calls were used for esv2676630 in the chromosome 16 conditional analyses (see Methods). (DOCX) [file pgen.1006760.s019.docx]

| **S14 Table.** Full models used for conditional analyses. | |
| --- | --- |
| **Trait** | **Model** |
|  | **† Full models used for genome-wide final round of conditional analysis** |
| RBC | RBC count ~ factor.sex + factor.CENTER + AGE + factor.CIGARETTE_USE + factor. genetic analysis group + log(sampling weight) + rs218265 + rs2032451 + rs9389268 + rs2075672 + esv2676630 + rs76539504 + rs855791+ rs7063597+ rs2798809 + rs141494605 + EV1 + EV2 + EV3 + EV4 + EV5 + (1\| census block group) + (1\| shared household) + (1\|relatedness_evs_all_noAsnOutliers)+ Genotype |
| HGB | HGB ~ factor.sex + factor.CENTER + AGE + factor.CIGARETTE_USE + factor. genetic analysis group + log(sampling weight) + logSquared(sampling weight) + rs17034641 + rs2032451 + esv2676630 + rs855791 + EV1 + EV2 + EV3 + EV4 + EV5 + (1\| census block group) + (1\| shared household) + (1\|relatedness) + Genotype |
| HCT | HCT ~ factor.sex + factor.CENTER + AGE + factor.CIGARETTE_USE + factor. genetic analysis group + log(sampling weight) + rs3754140 + rs17034641 + rs334 + rs855791 + EV1 + EV2 + EV3 + EV4 + EV5 + (1\| census block group) + (1\| shared household) + (1\|relatedness) + Genotype |
| MCV | MCV ~ factor.sex + factor.CENTER + AGE + factor.CIGARETTE_USE + factor. genetic analysis group + log(sampling weight) + rs218265 + rs2032451 + rs9389268 + rs141848064 + rs334 + esv2676630 + rs855791 + rs7063597 + rs4714548 + rs607203 + rs113342804 + rs141494605 + rs148323035 + rs28888111 + rs74001178 + EV1 + EV2 + EV3 + EV4 + EV5 (1\| census block group) + (1\| shared household) + (1\|relatedness) + Genotype |
| MCH | MCH ~ factor.sex + factor.CENTER + AGE + factor.CIGARETTE_USE + factor. genetic analysis group + log(sampling weight) + logSquared(sampling weight) + rs12634180 + rs218265 + rs2032451 + rs9389268 + rs607203 + esv2676630 + rs855791 + rs7063597 + rs9367125 + rs141494605 + rs145546625 + rs28888111 + rs74001178 + EV1 + EV2 + EV3 + EV4 + EV5 + (1\| census block group) + (1\| shared household) + Genotype |
| MCHC | MCHC ~ factor.sex + factor.CENTER + AGE + factor.CIGARETTE_USE + factor. genetic analysis group + log(sampling weight) + logSquared(sampling weight) + rs1349471 + rs334 + esv2676630 + rs551118 + rs855791 + rs141494605 + EV1 + EV2 + EV3 + EV4 + EV5 + (1\| census block group) + (1\| shared household) + (1\|relatedness) + Genotype |
| RDW | Log(RDW) ~ factor.sex + factor.CENTER + AGE + factor.CIGARETTE_USE + factor. genetic analysis group + log(sampling weight) + logSquared(sampling weight) + rs6685034 + rs4975528 + rs6864465 + rs17764730 + rs941718 + rs111473449 + esv2676630 + rs855791 + rs7063597 + EV1 + EV2 + EV3 + EV4 + EV5 + (1\| census block group) + (1\| shared household) + (1\|relatedness) + Genotype |
|  | **†† Full models used for conditional analysis at the 16p13.3 locus using the probe intensity-based calls for the alpha gene CNV** |
| RBC | RBC count ~ factor.sex + factor.CENTER + AGE + factor.CIGARETTE_USE + factor. genetic analysis group + log(sampling weight) + hba_cnv_countDel + hba_cnv_countDupl + EV1 + EV2 + EV3 + EV4 + EV5 + (1\| census block group) + (1\| shared household) + (1\|relatedness_evs_all_noAsnOutliers)+ Genotype |
| HGB | HGB ~ factor.sex + factor.CENTER + AGE + factor.CIGARETTE_USE + factor. genetic analysis group + log(sampling weight) + logSquared(sampling weight) + hba_cnv_countDel + hba_cnv_countDupl + EV1 + EV2 + EV3 + EV4 + EV5 + (1\| census block group) + (1\| shared household) + (1\|relatedness) + Genotype |
| HCT | HCT ~ factor.sex + factor.CENTER + AGE + factor.CIGARETTE_USE + factor. genetic analysis group + log(sampling weight) hba_cnv_countDel + hba_cnv_countDupl + EV1 + EV2 + EV3 + EV4 + EV5 + (1\| census block group) + (1\| shared household) + (1\|relatedness) + Genotype |
| MCV | MCV ~ factor.sex + factor.CENTER + AGE + factor.CIGARETTE_USE + factor. genetic analysis group + log(sampling weight) + hba_cnv_countDel + hba_cnv_countDupl + rs148323035 + EV1 + EV2 + EV3 + EV4 + EV5 (1\| census block group) + (1\| shared household) + (1\|relatedness) + Genotype |
| MCH | MCH ~ factor.sex + factor.CENTER + AGE + factor.CIGARETTE_USE + factor. genetic analysis group + log(sampling weight) + logSquared(sampling weight) + hba_cnv_countDel + hba_cnv_countDupl + rs145546625 + EV1 + EV2 + EV3 + EV4 + EV5 + (1\| census block group) + (1\| shared household) + Genotype |
| MCHC | MCHC ~ factor.sex + factor.CENTER + AGE + factor.CIGARETTE_USE + factor. genetic analysis group + log(sampling weight) + logSquared(sampling weight) + hba_cnv_countDel + hba_cnv_countDupl + EV1 + EV2 + EV3 + EV4 + EV5 + (1\| census block group) + (1\| shared household) + (1\|relatedness) + Genotype |
| RDW | Log(RDW) ~ factor.sex + factor.CENTER + AGE + factor.CIGARETTE_USE + factor. genetic analysis group + log(sampling weight) + logSquared(sampling weight) + hba_cnv_countDel + hba_cnv_countDupl + EV1 + EV2 + EV3 + EV4 + EV5 + (1\| census block group) + (1\| shared household) + (1\|relatedness) + Genotype |
